# UMAD1 contributes to ESCRT-III dynamic subunit turnover during cytokinetic abscission

**DOI:** 10.1242/jcs.261097

**Published:** 2023-08-10

**Authors:** James Glover, Edward J. Scourfield, Leandro N. Ventimiglia, Xiaoping Yang, Steven Lynham, Monica Agromayor, Juan Martin-Serrano

**Affiliations:** ^1^ Department of Infectious Diseases, King's College London, Faculty of Life Sciences & Medicine, London SE1 9RT, UK; ^2^ Proteomics Facility, Centre of Excellence for Mass Spectrometry, King's College London, London SE5 9NU, UK

**Keywords:** Cytokinesis, Midbody, ESCRT, Membrane remodelling

## Abstract

Abscission is the final stage of cytokinesis whereby the midbody, a thin intercellular bridge, is resolved to separate the daughter cells. Cytokinetic abscission is mediated by the endosomal sorting complex required for transport (ESCRT), a conserved membrane remodelling machinery. The midbody organiser CEP55 recruits early acting ESCRT factors such as ESCRT-I and ALIX (also known as PDCD6IP), which subsequently initiate the formation of ESCRT-III polymers that sever the midbody. We now identify UMAD1 as an ESCRT-I subunit that facilitates abscission. UMAD1 selectively associates with VPS37C and VPS37B, supporting the formation of cytokinesis-specific ESCRT-I assemblies. TSG101 recruits UMAD1 to the site of midbody abscission, to stabilise the CEP55–ESCRT-I interaction. We further demonstrate that the UMAD1–ESCRT-I interaction facilitates the final step of cytokinesis. Paradoxically, UMAD1 and ALIX co-depletion has synergistic effects on abscission, whereas ESCRT-III recruitment to the midbody is not inhibited. Importantly, we find that both UMAD1 and ALIX are required for the dynamic exchange of ESCRT-III subunits at the midbody. Therefore, UMAD1 reveals a key functional connection between ESCRT-I and ESCRT-III that is required for cytokinesis.

## Teaser

**Highlighted Article:** UMAD1 is identified as a novel component of the ESCRT machinery, and specifically facilitates cytokinetic abscission. UMAD1 plays an unexpected role in the dynamic exchange of membrane remodelling complexes at the midbody.

## INTRODUCTION

Cytokinetic abscission completes cell division by resolving the midbody, a thin intercellular bridge that remains as a physical connection between emerging daughter cells (Mierzwa and Gerlich, 2014). Actin remodelling and microtubule severing are critical events preceding abscission (Addi et al., 2018) and midbody cleavage is ultimately facilitated by the endosomal sorting complex required for transport (ESCRT) machinery (Carlton and Martin-Serrano, 2007; Elia et al., 2011; Guizetti et al., 2011; Morita et al., 2007). ESCRT-III subunits form filaments at the inner face of cytosol-filled membranous stalks to catalyse bending and scission of membranes away from the cytoplasm (Caillat et al., 2019; McCullough et al., 2018; Pfitzner et al., 2021). This process requires the AAA ATPase VPS4 (which has VPS4A and VPS4B paralogues in mammals), which provides the driving force to dynamically reshape and ultimately disassemble ESCRT-III polymers (Azad et al., 2023; Caillat et al., 2019; Mierzwa et al., 2017). The ability to constrict and sever membranes underpins the fundamental and highly conserved role of the ESCRT machinery in topologically related processes, such as multivesicular body formation, enveloped virus budding, membrane repair, mitotic resealing of the nuclear envelope and pruning of neurons (Gatta and Carlton, 2019; McCullough et al., 2018; Migliano et al., 2022; Schöneberg et al., 2017; Scourfield and Martin-Serrano, 2017).

The broad functionality of the ESCRT machinery requires a striking adaptability at multiple levels, including site-specific adapters that recruit ESCRT-III at different cellular locations. Thus, the midbody organiser CEP55 orchestrates abscission by recruiting the ESCRT-associated protein ALIX (also known as PDCD6IP) and the ESCRT-I subunit TSG101 to the site of abscission, initiating two parallel pathways that nucleate ESCRT-III filament formation (Carlton and Martin-Serrano, 2007; Lee et al., 2008; Morita et al., 2007). A direct association between ALIX and the core component of ESCRT-III, CHMP4B, is required for abscission (Carlton et al., 2008). Alternatively, the sequential interaction of CEP55 with TSG101, ESCRT-II and CHMP6, provides a second pathway to recruit ESCRT-III to the midbody (Christ et al., 2016). The dynamic exchange of ESCRT-III subunits by VPS4 underpins the formation of larger ESCRT-III assemblies that are required for membrane scission (Mierzwa et al., 2017).

The role of ESCRT proteins in cell division is conserved from Archaea to eukaryotes, yet a high level of plasticity in this function has been revealed. The archaeal CdvA adapter recruits ESCRT-III during cell division (Samson et al., 2011) whereas midbody recruitment of ALIX and TSG101 in *Drosophila*, which lack a CEP55 homologue, is facilitated by their direct interaction with the homologue of the midbody component MKLP1 (Lie-Jensen et al., 2019). More recent work in mice has shown a restricted role of CEP55 in rapidly dividing cells such as neuronal stem cells (Little et al., 2021; Tedeschi et al., 2020), suggesting either tissue specific recruiters of ESCRT-III or that parallel abscission pathways exist that can compensate for ESCRT deficiencies.

To better understand functional adaptations and plasticity in the ESCRT machinery we have focussed this study on ESCRT-I, a heterotetramer that in mammalian cells contains TSG101, VPS28, one of four VPS37 subunits (VPS37A, VPS37B, VPS37C or VPS37D) and one of at least three MVB12-like subunits (MVB12A, MVB12B or UBAP1) (Flower et al., 2020; Gill et al., 2007; Kostelansky et al., 2007). A well-characterised selective subunit pairing has been described between VPS37A and UBAP1, which form a ubiquitin-binding ESCRT-I module that is highly adapted to endosomal sorting of ubiquitylated cargo and autophagosome closure (Agromayor et al., 2012; Wunderley et al., 2014). However, specific roles and selective pairing preferences for other ESCRT-I modules remain unclear.

Here, we identify UBAP1-MVB12-associated domain containing 1 (UMAD1) as a novel MVB12-like subunit of ESCRT-I. UMAD1 is incorporated into ESCRT-I through its ability to interact with TSG101, and VPS37B or VPS37C, via its conserved UMA domain. Importantly, UMAD1 is required to stabilise the CEP55–TSG101 interaction and UMAD1-depleted cells show cytokinetic defects that are synergistic with ALIX co-depletion. UMAD1 is a key component of the cytokinetic pathway that reveals an unexpected connection between early acting ESCRT factors and the dynamic exchange of ESCRT-III at the midbody.

## RESULTS

### UMAD1 is a novel MVB12-like subunit which selectively binds VPS37B or VSP37C through its UMA domain

**Fig. 1.**
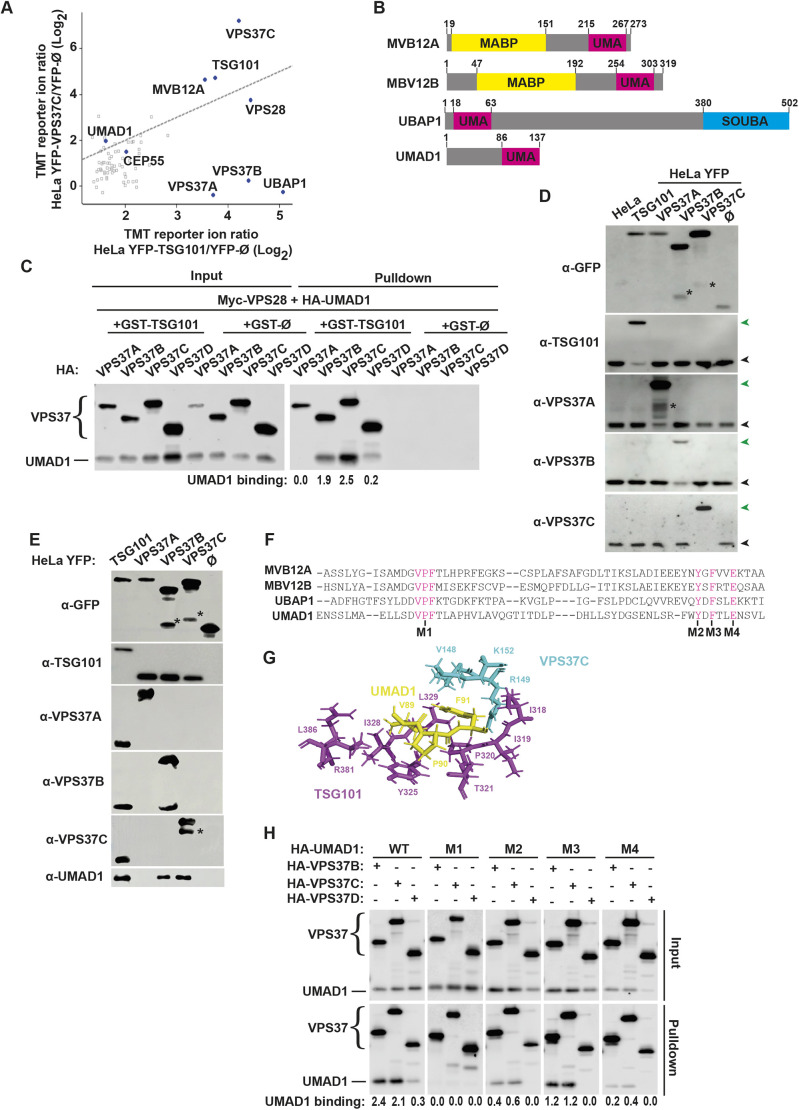
**UMAD1 binds ESCRT-I through its UMA domain.** (A) IP-TMT mass spectrometry-based analysis of ESCRT-I subunit enrichment in YFP–TSG101 and YFP–VPS37C-containing complexes. Scatter plot of one representative experiment showing TMT values obtained for YFP–TSG101 and YFP–VPS37C as abundance ratios over background values from unfused YFP (YFP-Ø) control. Each dot represents a protein measurement. The diagonal line represents a function of *y*=*x* that corresponds to similar enrichment for both baits. (B) Schematic diagram showing the domain structure of human MVB12-like proteins. MABP, matrix-associated β-prism; UMA, UBAP1-MVB12-associated domain; UBA, ubiquitin-associated domain; SOUBA, solenoid of overlapping UBA domains. (C) Co-precipitation assays using transiently overexpressed tagged ESCRT-I subunits. 293T cells were co-transfected with GST–TSG101, Myc–VPS28, HA–UMAD1 and HA-tagged VPS37A, VPS37B, VPS37C or VPS37D. Cell lysates (input, 1%), as well as the glutathione-bound fraction (pulldown) were analysed by blotting with anti-HA antibody. Unfused GST (GST-Ø) was used as negative control. UMAD1-binding values represent the UMAD1 band intensity in the pulldown divided by the input band intensity. (D) Western blots of HeLa cells stably expressing YFP–TSG101, YFP–VPS37A, YFP–VPS37B YFP–VPS37C or unfused YFP (YFP-Ø). Membranes were incubated with antibodies against the indicated ESCRT-I proteins or GFP. Green arrowheads indicate the expected position of YFP-tagged protein; black arrowheads indicate the untagged endogenous protein. For the YFP-Ø input, one tenth of the sample was loaded for the blot with anti-GFP antibody. (E) Affinity purification using HeLa cells stably expressing near-physiological levels of YFP-tagged TSG101, VPS37A, VPS37B or VPS37C. The YFP-bound fraction (pulldown) was analysed by western blotting with anti-GFP or -UMAD1 antibodies or antibodies against each of the indicated ESCRT-I proteins. YFP-Ø was used as control bait. Asterisks in D and E denote cleaved YFP fusion. For the YFP-Ø input, one-tenth of the sample was loaded for the blot with anti-GFP antibody. (F) Sequence alignment of the UMA domains of MVB12A, MVB12B, UBAP1 and UMAD1, showing the position of the alanine substitutions introduced to generate UMAD mutants (V_89_P_90_F_91_/AAA, Y125A, F127A, E130A; UMAD^M1^ to UMAD^M4^, respectively). (G) Close-up representation of AlphaFold2 prediction of the binding interface between TSG101, VPS37C and UMAD1. TSG101, magenta; VPS37, cyan; VPF motif (UMAD1), yellow. See also Fig. S1. (H) GST pulldown assay showing UMAD1 incorporation into ESCRT-I complexes containing VPS37B, VPS37C or VPS37D through its UMA domain. 293T cells were co-transfected with GST–TSG101, Myc–VPS28, HA-tagged VPS37B, VPS37C or VPS37D and HA-tagged wild-type UMAD1 or each of the UMAD1 mutants shown in F. Cell lysates (input, 1%), as well as the glutathione-bound fraction (pulldown) were analysed by blotting with anti-HA antibody. UMAD1 binding shows the UMAD1 band intensity in the pulldown divided by the input band intensity. All data representative of at least three repeats.

To identify novel ESCRT-I-interacting partners, we generated HeLa cells stably expressing either YFP–TSG101 or YFP–VPS37C at near physiological levels. Affinity purification of YFP fusion proteins was performed using a GFP-trap approach, whereby stable expression of unfused YFP (YFP-Ø) served as a negative control so that the purified protein fractions were analysed by quantitative immunoprecipitation tandem mass tag (IP-TMT)-based mass spectrometry (MS) to identify hits enriched over YFP-Ø (Fig. 1A). A TMT tag reporter ion ratio ≥2 for YFP-TSG101:YFP-Ø or YFP-VPS37C:YFP-Ø defined specific associations. As a validation of this approach, core components of ESCRT-I such as VPS28 and MVB12A were enriched above this threshold in both YFP–TSG101 and YFP–VPS37C baits. ESCRT-I subunit selectivity was demonstrated by the lack of enrichment of endogenous VPS37A and VPS37B in the YFP–VPS37C bait, as the incorporation of VPS37 subunits is thought to be mutually exclusive. Conversely, the enrichment of VPS37A, VPS37B and VPS37C by the YFP–TSG101 bait is fully consistent with the incorporation of TSG101 in all possible ESCRT-I assemblies. Besides ESCRT-I subunits, an interaction with CEP55 was observed with both baits, thus indicating that YFP–TSG101 and YFP–VPS37C recapitulate physiological ESCRT-I interactions. In addition to known ESCRT-I subunits, UMAD1 was identified as a potential interaction partner for both YFP–TSG101 and YFP–VPS37C (Fig. 1A). UMAD1 is conserved across vertebrates, and data from the Human Protein Atlas suggest ubiquitous expression (Uhlén et al., 2015). Interestingly, a bioinformatics study identified a putative UMA domain in UMAD1 (Fig. 1B) that is conserved among MVB12-like subunits (de Souza and Aravind, 2010), which might promote its association with ESCRT-I.

Specific incorporation of UMAD1 into ESCRT-I and subunit pairing specificity was first explored using a GST co-precipitation assay. GST–TSG101, Myc–VPS28 and HA–UMAD1 were co-transfected with either HA–VPS37A, HA–VPS37B, HA–VPS37C or HA–VPS37D (Fig. 1C). This assay confirmed UMAD1 incorporation into ESCRT-I with a pairing preference for complexes containing VPS37C followed by VPS37B. A marginal association of UMAD1 was detected with VPS37D, whereas no incorporation was observed with VPS37A. A potential caveat with this experimental approach is that overexpression of ESCRT-I subunits might alter pairing selectivity compared to physiological expression levels. We therefore took advantage of HeLa cells stably expressing YFP-tagged TSG101, VPS37A, VPS37B or VPS37C. Importantly, the stable expression of exogenous VPS37 fusions replaced the corresponding endogenous proteins to achieve near physiological levels (Fig. 1D), a phenomenon that resembles the regulation of TSG101 levels by the ‘steadiness box’ to ensure that physiological levels of ESCRT-I subunits are maintained (Feng et al., 2000; McDonald and Martin-Serrano, 2008). Western blot analysis of the YFP-affinity-purified fractions showed the co-precipitation of TSG101 by each of the VPS37 baits, as expected (Fig. 1E). Importantly, the association of endogenous UMAD1 with YFP–TSG101 was confirmed, and this co-precipitation assay recapitulated the preferential pairing of endogenous UMAD1 with VPS37C, followed by a reduced association with VPS37B (Fig. 1E). Together with the MS results, these observations demonstrate that UMAD1 is a genuinely expressed protein that is incorporated into a subset of ESCRT-I heterotetramers, with preference for those containing VPS37C.

Given the established role of UMA domains in ESCRT-I binding, we performed site-directed mutagenesis on residues in this area that are conserved with MVB12A, MVB12B and UBAP1(de Souza and Aravind, 2010) (Fig. 1F). The resulting UMAD1 mutants [the triple mutant V89A, P90A and F91A (V_89_P_90_F_91_/AAA), Y125A, F127A and E130A; denoted UMAD^M1^ to UMAD^M4^, respectively] were tested by co-precipitation assays as described above, revealing that mutation of the signature VPF motif (UMAD1^M1^) completely abolished binding to ESCRT-I (Fig. 1H). This observation is entirely consistent with the direct contacts made between the VPF motif in MVB12A with a hydrophobic pocket formed by TSG101 and VPS37B (Flower et al., 2020). Importantly, the AlphaFold2 prediction for the structure of a UMAD1-containing ESCRT-I headpiece supports conserved contacts between TSG101, VPS37C and the VPF motif in UMAD1 (Fig. 1G; Fig. S1). Together these findings demonstrate that UMAD1 is a novel alternative MVB12-like subunit that interacts with ESCRT-I through its UMA domain.

### UMAD1 stabilises the CEP55–TSG101 interaction

**Fig. 2.**
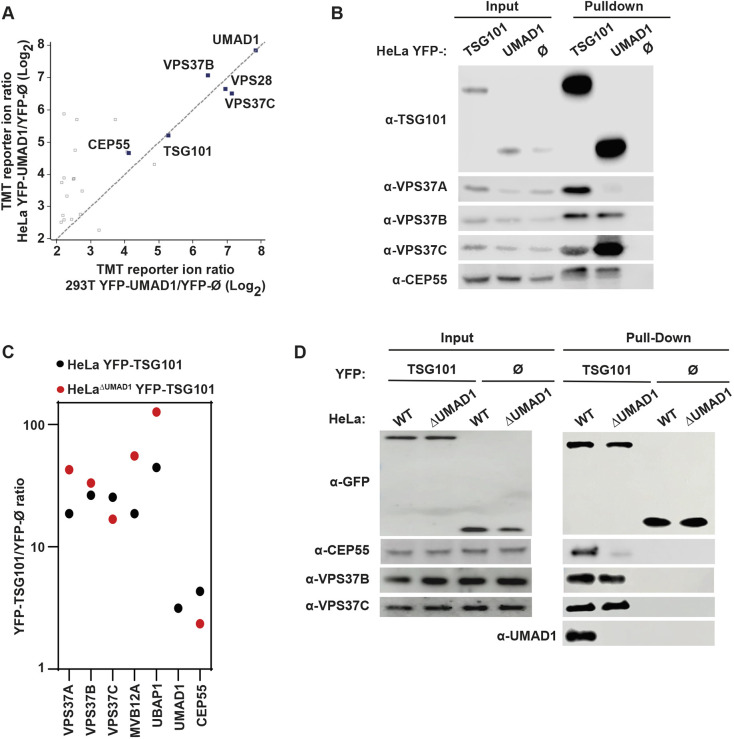
**UMAD1 stabilises the CEP55/TSG101 interaction.** (A) Plot showing UMAD1 interaction partners identified by IP-TMT-based mass spectrometry using YFP–UMAD1 stably re-expressed in HeLa^ΔUMAD1^ and 293T^ΔUMAD1^ cells as bait. Each dot represents a protein measurement, and the diagonal line represents a function of *y=x*. (B) GFP-trap immunoprecipitation using HeLa^ΔUMAD1^ cells stably re-expressing YFP–UMAD1. Cell lysates (input, 1%) and the GFP-bound fraction (pulldown) were analysed by western blotting with antibodies against each of the indicated proteins. HeLa cells that stably express YFP–TSG101 or unfused YFP (YFP-Ø) were used as positive and negative controls, respectively. (C) IP-TMT-based mass spectrometry was used to determine differences in ESCRT-I subunit enrichment after co-precipitation of YFP–TSG101 stably expressed in either HeLa^WT^ or HeLa^ΔUMAD1^ cells. Fold enrichment of ESCRT-I proteins in the HeLaΔUMAD1 versus the HeLa^WT^ background is shown. (D) Pulldown experiments using HeLa or HeLa^ΔUMAD1^ cells that stably express YFP–TSG101 or unfused YFP (YFP-Ø). Cell lysates (input, 1%) and affinity purified proteins (pulldown) were analysed by western blot using anti-GFP, anti-CEP55, anti-VPS37B or anti-VPS37C antibodies. Pulldowns were also analysed with an anti-UMAD1 antibody. For the YFP-Ø input, one-tenth of the sample was loaded for the blot with anti-GFP antibody. See also Fig. S2. All data representative of at least three repeats.

To identify and validate UMAD1 interaction partners, a reciprocal IP-TMT-based MS screen was performed. Briefly, the CRISPR/Cas9 system was used to generate *UMAD1-*knockout cells (HeLa^ΔUMAD1^ and 293T^ΔUMAD1^) (Fig. S2), which were subsequently transduced with retroviruses to stably express YFP– UMAD1 (HeLa^ΔUMAD1/YFP-UMAD1^ and 293T^ΔUMAD1/YFP-UMAD1^) to enable affinity purification without interference from endogenous UMAD1. Analysis of the bound fraction in both cell lines confirmed association of YFP–UMAD1 with the endogenous core ESCRT-I subunits, TSG101 and VPS28. In contrast, ESCRT-II subunits were not detected in these experiments, thus highlighting the selective association of UMAD1 with ESCRT-I. In agreement with the selective subunit pairing, VPS37C and VPS37B associated with YFP–UMAD1 whereas VPS37A, MVB12A and UBAP1 were not enriched above the threshold (Fig. 2A). Interestingly, CEP55 was also identified as a UMAD1 interaction partner in both HeLa^ΔUMAD1/YFP-UMAD1^ and 293T^ΔUMAD1/YFP-UMAD1^ cells (Fig. 2A). To validate these observations, a comparative co-precipitation of HeLa^WT/YFP-TSG101^ and HeLa^ΔUMAD1/YFP-UMAD1^ was performed (Fig. 2B). Endogenous TSG101, VPS37B and VPS37C, but not VPS37A, were co-precipitated by YFP–UMAD1. Intriguingly, HeLa^WT/YFP-TSG101^ and HeLa^ΔUMAD1/YFP-UMAD1^ showed co-precipitation of endogenous CEP55 at comparable levels. We therefore reason that UMAD1 might form an ESCRT-I module that selectively binds CEP55. Intriguingly, the CEP55 bound to UMAD1 runs at higher molecular mass than the CEP55 in the input fraction. Given that CEP55 is regulated by phosphorylation (Bastos and Barr, 2010), we speculate that UMAD1 might bind preferentially to the phosphorylated form of CEP55, but this needs to be further investigated.

To determine the impact of UMAD1 on endogenous ESCRT-I composition, we performed an IP-TMT-based MS screen using stably expressed YFP–TSG101, in either HeLa^WT^ or HeLa^ΔUMAD1^ cells, as bait. As expected from the results above, UMAD1 was enriched in the HeLa^WT/YFP-TSG101^ eluates over the YFP-Ø background whereas it was undetectable in HeLa^ΔUMAD1/YFP-TSG01^ cells (Fig. 2C). Importantly, the ratios for UBAP1 and MVB12A were increased in the HeLa^ΔUMAD1^ background as compared to those in HeLa^WT^ cells, supporting the mutually exclusive incorporation of MVB12-like subunits into ESCRT-I. In addition, the selective pairing of VPS37 subunits with UMAD1 was further confirmed. Thus, whereas the signal for VPS37B displayed a slight reduction in the HeLa^ΔUMAD1^ background, a greater decrease was observed for VPS37C, consistent with the pairing preference of UMAD1 with VPS37C. Conversely, the VPS37A and UBAP1 ratios were significantly increased in the UMAD1-knockout background, suggesting that UMAD1 competes with UBAP1 for ESCRT-I association. Finally, the CEP55 signal was markedly decreased in the HeLa^ΔUMAD1^ background, and this reduced binding was confirmed by western blot analysis of the corresponding bound fractions (Fig. 2D). Altogether, this data indicates that UMAD1 stabilises the CEP55–TSG101 interaction, suggesting that it has potential roles in cytokinesis.

### UMAD1 facilitates cytokinetic abscission

**Fig. 3.**
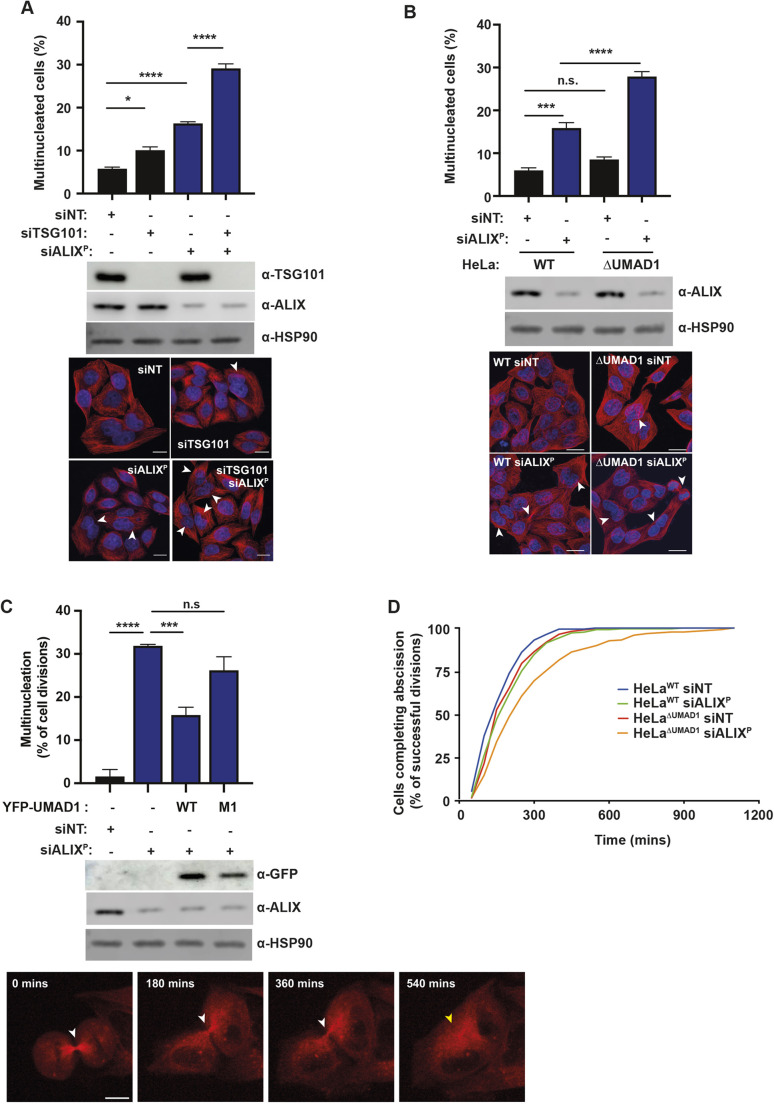
**UMAD1 is required for cytokinetic abscission.** (A) Quantification of multinucleation in HeLa cells upon siRNA treatment as indicated. Error bars are mean±s.e.m.; *n*≥300 cells from three independent experiments. Full depletion of TSG101 and partial depletion of ALIX (siALIX^P^) is shown by the western blot. HSP90 levels serve as loading control. Representative confocal images from cells as above. (B) Quantification of multinucleation in HeLa^WT^ and HeLa^ΔUMAD1^ cells upon partial depletion of ALIX (siALIX^P^). Error bars are mean±s.e.m.; *n*≥300 cells from three independent experiments.). Representative confocal images and western blots showing ALIX depletion as above. For A and B, white arrowheads in confocal images highlight multinucleated cells. *P<0.05; ***P<0.001; ****P<0.0001; n.s., not significant (unpaired one-way ANOVA with Tukey post-hoc multiple comparisons). Scale bars: 15 µm. (C) Asynchronous cultures of HeLa^ΔUMAD1^ cells stably expressing mCherry–tubulin alone or in combination with YFP–UMAD wild-type (WT) or ESCRT-I binding mutants (M1) were analysed by live-cell microscopy upon siRNA treatment as indicated. Graph shows the quantification of cells that become multinucleated after abscission failure. Error bars are mean±s.e.m.; *n*≥78 cells per condition from three independent experiments; ***P<0.001; ****P<0.0001; n.s., not significant (unpaired one-way ANOVA with Tukey post-hoc multiple comparisons). Representative confocal images and western blots showing ALIX depletion as above. White arrowheads indicate midbodies, yellow arrowhead indicates cytokinetic failure. Scale bar: 10 µm. (D) Asynchronous cultures of HeLa^WT^ and HeLa^ΔUMAD1^ cells stably expressing mCherry–tubulin were analysed by live-cell microscopy upon siRNA treatment as indicated. Graph shows the time interval between furrow ingression and abscission in cells that successfully complete cytokinesis; *n*≥250 cells scored per condition from three independent experiments. The abscission times (mean±s.e.m.) are as follows: HeLa^WT^+siNT, 178.0±4.8 min; HeLa^WT^+siALIX^P^, 216.9±7.1 min; HeLa^ΔUMAD1^+siNT, 206.1±5.4 min; HeLa^ΔUMAD1^+siALIX^P^, 298.3±12.8 min. A Kruskal–Wallis test was performed to determine the following *P*-values: HeLa^WT^+siNT versus HeLa^WT^+siALIX^P^=0.0002; HeLa^WT^+siNT versus HeLa^ΔUMAD1^+siNT=0.0018; HeLa^WT^+siALIX^P^ versus HeLa^ΔUMAD1^+siNT=>0.9999; HeLa^ΔUMAD1^+siALIX^P^ versus all others ≤0.0001. See also Fig. S3 and Movie 2. All data representative of at least three repeats.

The initial characterisation of HeLa^ΔUMAD1^ cells showed a 4-fold reduction in clonogenic growth compared to HeLa^WT^ cells (Fig. S3A), a phenotype consistent with a role of UMAD1 in the completion of cell division. We then examined markers for cytokinesis failure in UMAD1-deficient cells and observed that levels of multinucleation were modestly increased at steady state in HeLa^ΔUMAD1^ versus HeLa^WT^ cells (Fig. S3B). We reasoned that this mild multinucleation phenotype could be due to compensation by ALIX, as this ESCRT-III nucleation axis is dominant over ESCRT-I (Carlton et al., 2008; Carlton and Martin-Serrano, 2007; Christ et al., 2016). Consistently with this model, depletion of TSG101 in the HeLa^WT^ background resulted in a 1.7-fold increase in multinucleation compared with a non-targeting control (Fig. 3A), resembling the cytokinetic defects in HeLa^ΔUMAD1^ cells. To further examine the synergistic effects between the two abscission arms, we titrated the concentration of ALIX-specific siRNA needed to achieve partial depletion of the protein in HeLa^WT^ cells so that the full extent of the synergistic phenotype could be unveiled (Fig. S3C). We observed that partial reduction of ALIX to 30% of normal protein levels (siALIX^P^) increased multinucleation, and further cytokinetic failure was observed upon TSG101 co-depletion (Fig. 3A). Critically, these synergistic rates of multinucleation were fully recapitulated by partially depleting ALIX in the HeLa^ΔUMAD1^ background (Fig. 3B). Similar synergistic effects were observed upon depletion of UMAD1 by siRNA (Fig. S3D,E), thus excluding the possibility that they were caused by off-target and clonal effects in HeLa^ΔUMAD1^ cells. We then studied the time of cytokinesis failure by live-cell microscopy in cells stably expressing mCherry–tubulin. We observed that HeLa^ΔUMAD1^ cells treated with siALIX^P^ became multinucleated after midbodies persisted for a long time (Fig. S3F; Movie 1), a phenotype consistent with abscission failure. We therefore used this system to address the cytokinetic role of the UMAD1–ESCRT-I interaction by performing functional rescue of HeLa^ΔUMAD1^ cells by re-expressing YFP-tagged UMAD1. As a control, the stable expression of YFP–UMAD1 wild-type in these cells fully rescued the cytokinetic effects associated with siALIX^P^ treatment, as the level of multinucleation was comparable to that in siALIX^P^-treated Hela^WT^ cells (Fig. 3A,C). Crucially, the VPF motif mutation in UMAD1 (YFP–UMAD1^M1^) that selectively inhibits the interaction with ESCRT-I, also abrogated the cytokinetic activity of UMAD1, as shown by the failure to rescue multinucleation levels in siALIX^P^-treated HeLa^ΔUMAD1^ cells (Fig. 3C; Movie 2). Therefore, the UMAD1–ESCRT-I interaction is required for cytokinesis, and this interaction cooperates with ALIX.

**Fig. 4.**
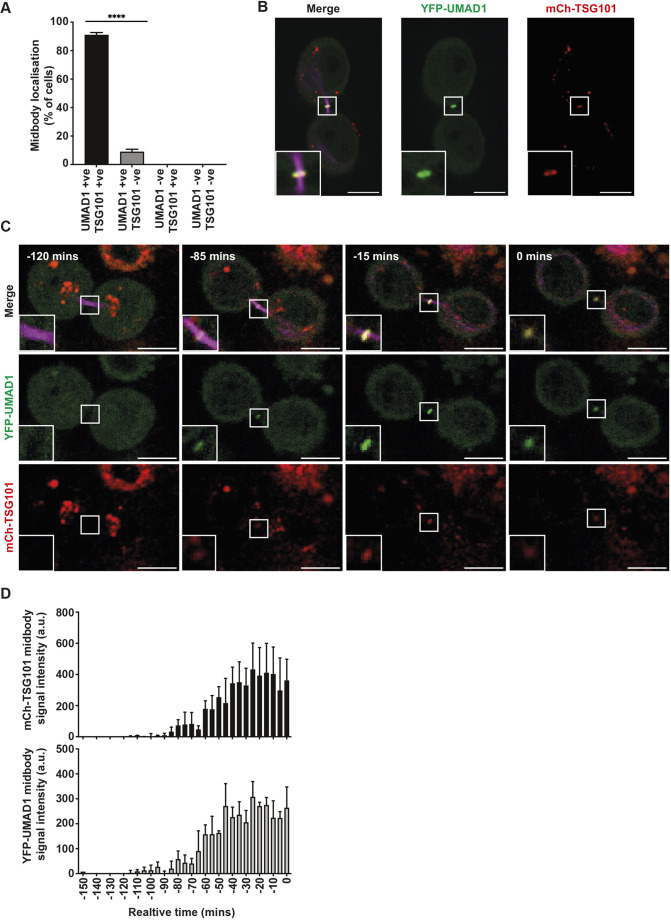
**TSG101 and UMAD1 colocalise at the midbody.** (A) Midbodies from HeLa^ΔUMAD1^ cells stably co-expressing YFP–UMAD1 and mCherry–TSG101 were scored live for the presence (+ve) or absence (−ve) of the fluorescent proteins. Error bars are mean±s.e.m.; *n*=45 midbodies from three independent experiments; ****P<0.0001 (unpaired two-tailed Student's *t*-test). (B) Representative confocal images of a cell scored in A showing UMAD1 (green) and TSG101 (red) colocalisation at the midbody (magenta). Insets show magnification of the selected area. Scale bars: 10 µm. (C,D) Live-cell analysis of YFP–UMAD1 and mCherry–TSG101 recruitment to the midbody over time. (C) Representative frames corresponding to time-lapse images of HeLa^ΔUMAD1^ cells stably co-expressing YFP–UMAD1 and mCherry–TSG101 at the indicated times during cytokinesis, where *t*=0 represents abscission. Insets show magnification of the selected areas. Scale bars: 10 µm. (D) Signal intensity plot of mCherry–TSG101 and YFP–UMAD1 at the midbody represented as arbitrary units (a.u.). Error bars are mean±s.e.m.; *n*=3 cells from three independent experiments. See also Movie 3. All data representative of at least three repeats.

**Fig. 5.**
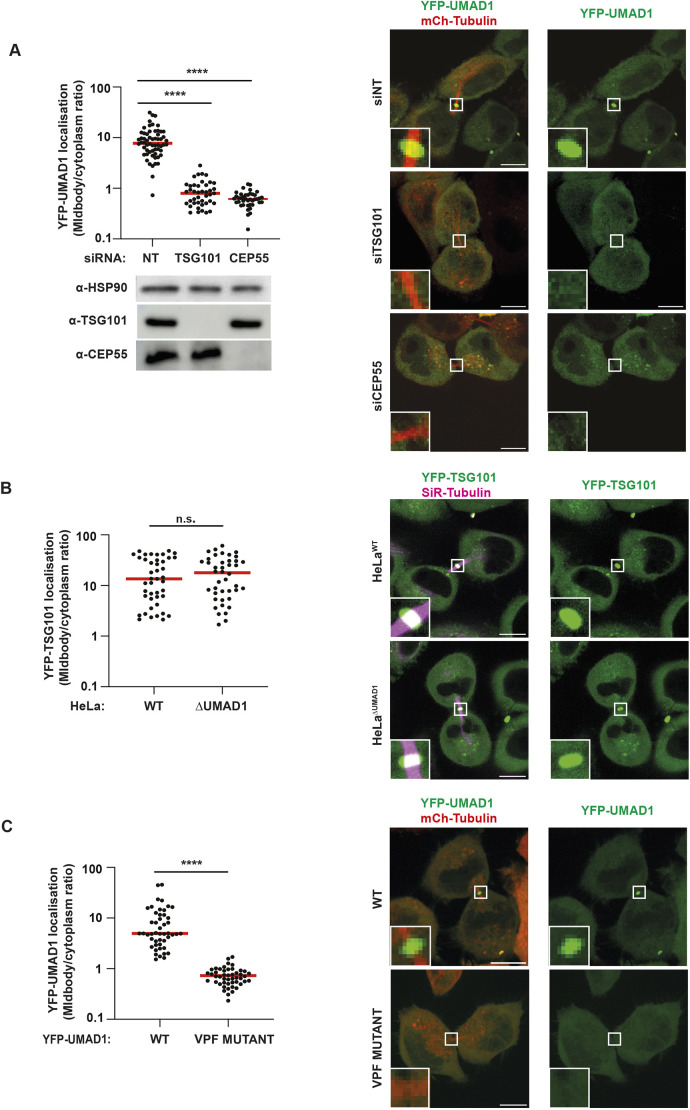
**UMAD1 recruitment to the midbody requires CEP55 and TSG101.** (A) Quantification of midbody localisation of YFP–UMAD in HeLa^ΔUMAD1^ cells stably co-expressing YFP–UMAD and mCherry–tubulin and treated with the indicated siRNAs. Red bars indicate mean values. siNT, *n*=60; siTSG101, *n*=43; siCEP55, *n*=40 cells from three independent experiments. ****P<0.0001 (unpaired one-way ANOVA test with Tukey post-hoc multiple comparisons). Depletion of TSG101 and CEP55 is shown by western blot. HSP90 levels serve as loading control. (B) Quantification of midbody localisation of YFP–TSG101 in HeLa^WT^ and HeLa^ΔUMAD1^ cells. Red bars indicate mean values. HeLa^WT^, *n*=43 cells; and HeLa^ΔUMAD1^, *n*=42 cells from three independent experiments. n.s., not significant, *P*>0.05 (unpaired two-tailed Student's *t*-test). (C) Quantification of midbody localisation of YFP–UMAD in HeLa^ΔUMAD1^ cells stably co-expressing either YFP–UMAD1 wild-type (WT) or mutated VPF motif (M1), and mCherry-tubulin. Red bars indicate mean values. YFP–UMAD1^WT^, *n*=48 cells; YFP–UMAD1^M1^, *n*=48 cells from three independent experiments. ****P<0.0001 (unpaired one-way ANOVA test with Tukey post-hoc multiple comparisons). Representative images are shown, where tubulin is pseudo-coloured in red and YFP–UMAD in green (A,C) and tubulin is pseudo-coloured in magenta and YFP–TSG101 in green (B). Insets show the midbody. Scale bars: 10 µm.

We next measured the impact of UMAD1 depletion on midbody resolution by live-cell microscopy (Fig. 3D). A modest abscission delay was observed in HeLa^ΔUMAD1^ cells, in agreement with the mild multinucleation phenotype in these cells. Supporting the role of UMAD1 in abscission, midbody resolution was severely delayed in HeLa^ΔUMAD1^ cells that were treated with siALIX^P^, whereas similar levels of ALIX depletion in HeLa^WT^ cells resulted in minor abscission delays. Given that YFP–UMAD1 is functional (Fig. 3C), we took advantage of this construct to investigate the localisation of UMAD1 during abscission. YFP–UMAD1 and mCherry–TSG101 were stably co-expressed in HeLa^ΔUMAD1^ cells, and live-cell microscopy revealed the complete colocalisation of both fluorescent proteins at the midbody (Fig. 4A,B). To determine the dynamics of YFP–UMAD1 and mCherry–TSG101 at the midbody, we tracked these cells from the early stages of cytokinesis to abscission. In agreement with previous reports, mCherry–TSG101 accumulated at the midbody during late stages of cytokinesis (Carlton and Martin-Serrano, 2007; Elia et al., 2011), and this localisation pattern was largely mirrored by YFP–UMAD1. Importantly, the (mCherry–TSG101)–(YFP–UMAD1) complex persisted until midbodies were resolved (Fig. 4C,D; Movie 3). Depletion of either TSG101 or CEP55 inhibited the recruitment of YFP–UMAD1 to the midbody (Fig. 5A), demonstrating that UMAD1 acts downstream of CEP55 and its recruitment to the midbody requires other ESCRT-I subunits. Conversely, YFP–TSG101 was recruited to late midbodies in both HeLa^WT^ and HeLa^ΔUMAD1^ cells (Fig. 5B), thus excluding defects in midbody recruitment of ESCRT-I as the cause of abscission delays in UMAD1-depleted cells. Finally, midbody localisation of YFP–UMAD1^M1^ was reduced to background levels (Fig. 5C), demonstrating that UMAD1 is recruited to the midbody by interacting with ESCRT-I. Altogether, these findings support that UMAD1 and TSG101 form an ESCRT-I module that is targeted to the midbody by CEP55, and the UMAD1–ESCRT-I interaction cooperates with ALIX to complete cytokinesis.

### Role of UMAD1–ESCRT-I in ESCRT-III dynamics at the midbody

**Fig. 6.**
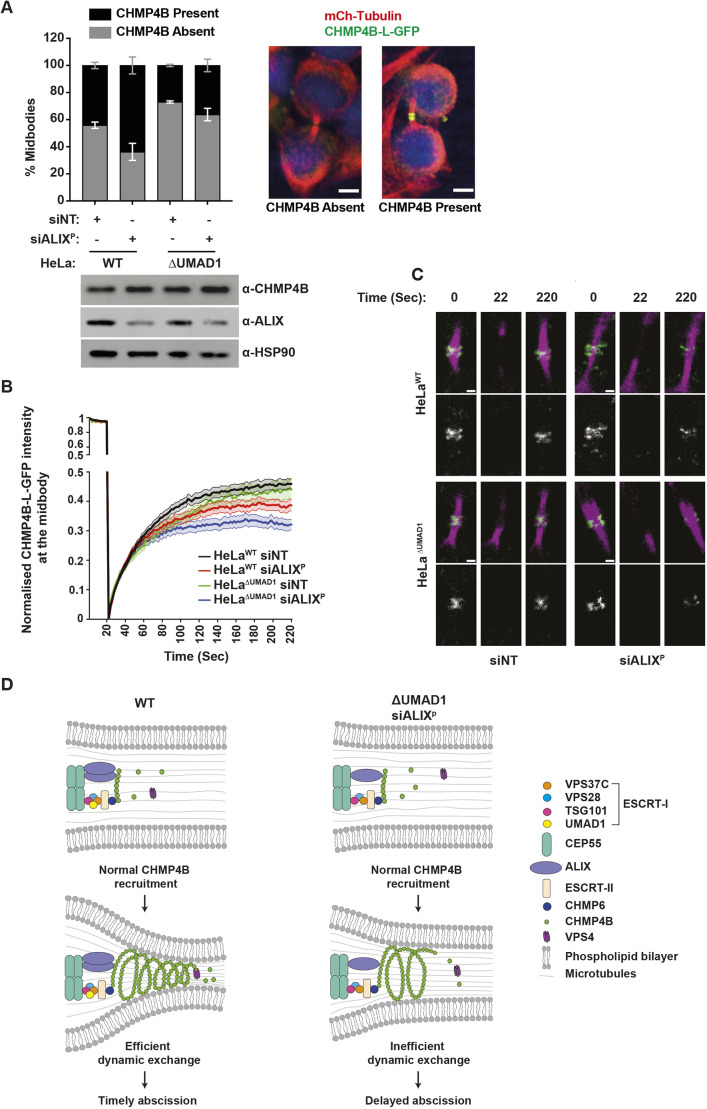
**UMAD1 determines the dynamic exchange of ESCRT-III at the midbody.** (A) Quantification of CHMP4B–L–GFP midbody localisation in HeLa^WT^ and HeLa^ΔUMAD1^ upon transfection with the indicated siRNA. Error bars are mean±s.e.m. HeLa+siNT, *n*=242; HeLa+siALIX, *n*=432; HeLaΔUMAD1+siNT, *n*=217; HeLaΔUMAD1+siALIX, *n*=270 from three independent experiments. Representative images of midbodies in HeLa cells stably expressing CHMP4B–L–GFP where the tagged protein is absent (left panel) or present (right panel) are shown. DNA is pseudo-coloured in blue, tubulin in red and CHMP4B–L–GFP in green. Scale bars: 5 µm. Cell lysates from siRNA-treated cells were analysed by western blot using the indicated antibodies. (B,C) FRAP of CHMP4B–L–GFP at HeLa^WT^ and HeLa^ΔUMAD1^ midbodies stained with SiR-tubulin following transfection with the indicated siRNA. (B) Fluorescence recovery curves of CHMP4B–L–GFP at the midbody. HeLa^WT^+siNT, *n*=34; HeLa^WT^+siALIX^P^, *n*=32; HeLa^ΔUMAD1^+siNT, *n*=20; HeLa^ΔUMAD1^+siALIX^P^, *n*=24 from three independent experiments (mean±s.e.m.). Statistical analysis was performed on the endpoint time=220 s. An ordinary one-way ANOVA with Tukey post-hoc multiple comparisons was performed to determine the following *P*-values: HeLa^WT^+siNT versus HeLa^WT^+siALIX^P^=0.0485; HeLa^ΔUMAD1^+siNT versus HeLa^ΔUMAD1^+siALIX^P^=0.0038; HeLa^WT^+siNT versus HeLa^ΔUMAD1^+siALIX^P^ *P*<0.0001; all other comparisons, *P*>0.05 (not significant). (C) Representative images of the photobleached region. First pre-bleach (*t*=0), bleach (*t*=22) and final post-bleach (*t*=220) frames are shown. CHMP4B–L–GFP (green) and SiR-tubulin (magenta) shown in top, CHMP4B–L–GFP (grey) shown in bottom. Scale bars: 1 µm. All data representative of at least three repeats. (D) Abscission model for HeLa^WT^ and HeLa^ΔUMAD1^ cells. In both backgrounds, CHMP4B recruitment is unperturbed. In HeLa^WT^ cells, there is efficient dynamic exchange of ESCRT-III and therefore timely abscission, whereas in HeLa^ΔUMAD1^ cells partially depleted of ALIX there is inefficient dynamic exchange which results in delayed abscission. See also Fig. S4.

Both ESCRT-I and ALIX promote the recruitment of ESCRT-III to the midbody at the end of cytokinesis (Elia et al., 2011; Guizetti et al., 2011). To answer whether *UMAD1* deficiency alters midbody recruitment of ESCRT-III, we took advantage of HeLa^WT^ and HeLa^ΔUMAD1^ cells stably expressing CHMP4B fused to a 25 nm flexible linker followed by GFP (CHMP4B–L–GFP), a functional marker for ESCRT-III that is expressed at sub-physiological levels (Fig. S4) (Ventimiglia et al., 2018). In HeLa^WT^ cells, CHMP4B–L–GFP localised to the midbody at steady state in 45% of cases, as expected from the recruitment of ESCRT-III during late stages of midbody resolution (Fig. 6A). The analysis of cells partially depleted of ALIX showed no reduction in CHMP4B–L–GFP recruitment to the midbody. In fact, a modest increase in the proportion of CHMP4B–L–GFP-positive midbodies was observed, suggesting the formation of abortive ESCRT-III polymers. Similarly, partial depletion of ALIX in HeLa^ΔUMAD1^ cells showed no significant impairment of CHMP4B–L–GFP recruitment to the midbody (Fig. 6A). Therefore, ESCRT-III recruitment defects do not underlie the synergy between UMAD1 and ALIX depletion in cytokinetic abscission. This puzzling result led us to explore the impact of ALIX and UMAD1 co-depletion on the dynamics of ESCRT-III at the midbody. For this purpose, we performed fluorescence recovery after photobleaching (FRAP) of CHMP4B–L–GFP at the midbody, as the turnover with a cytoplasmic pool allows ESCRT-III filament growth and membrane scission (Mierzwa et al., 2017). Strikingly, partial depletion of ALIX in HeLa^ΔUMAD1^ cells suppressed the turnover of CHMP4B–L–GFP at the midbody whereas a more modest reduction of fluorescence recovery was observed upon partial depletion of ALIX in HeLa^WT^ cells (Fig. 6B,C). Importantly the turnover of CHMP4B–L–GFP in control-treated cells was comparable between HeLa^WT^ and HeLa^ΔUMAD1^ cells. These observations are fully consistent with the abscission defects observed in cells co-depleted of UMAD1 and ALIX, thus supporting that UMAD1-containing ESCRT-I complexes and ALIX cooperate to sustain the net growth of ESCRT-III polymers during abscission.

## DISCUSSION

We have identified UMAD1 as a novel MVB12-like subunit that is incorporated into ESCRT-I by pairing preferentially with VPS37C. Importantly, we demonstrate that UMAD1-containing ESCRT-I heterotetramers have a specific role at the midbody to mediate cytokinetic abscission and cooperate with the ALIX-dependent arm of abscission.

We show that UMAD1 can integrate into ESCRT-I assemblies that contain either VPS37B or VPS37C, with a strong pairing preference for VPS37C. Besides the module containing UBAP1 and VPS37A, which is involved in endosomal sorting (Agromayor et al., 2012; Stefani et al., 2011), this represents the second known example of a selective ESCRT-I subunit pairing that specifically facilitates a cellular function. It has been proposed that MVB12A associates with VPS37C (Wunderley et al., 2014), but this pairing was observed under conditions that reduce the specificity of ESCRT-I assemblies. Nonetheless, we cannot exclude that multiple subsets of VPS37C-containing complexes may co-exist. Alternatively, the VPS37C–UMAD1 pairing might be favoured during mitosis to facilitate cytokinetic abscission.

We demonstrate that UMAD1 requires its signature VPF motif at the UMA domain to facilitate its incorporation into ESCRT-I at the midbody, and the UMAD1–ESCRT-I interaction is also required for the completion of cytokinesis. We further show that UMAD1 stabilises the interaction between CEP55 and TSG101, but how this is achieved requires further study. UMAD1 could provide additional contacts between the proline-rich region in TSG101 and the ESCRT and ALIX-binding region (EABR) of CEP55 (Carlton and Martin-Serrano, 2007; Lee et al., 2008; Morita et al., 2007). Alternatively, incorporation of UMAD1 into ESCRT-I could induce structural changes in TSG101 to promote the interaction with CEP55.

Importantly, UMAD1 and TSG101 colocalise at the midbody during the final stages of cytokinesis and UMAD1 depletion delays midbody resolution, increases cytokinetic defects and perturbs ESCRT-III dynamics at the midbody. Altogether, these results support that UMAD1 forms an abscission competent ESCRT-I module that is recruited by CEP55. As the cytokinetic defects observed in the absence of UMAD1 were recapitulated by TSG101 depletion, we propose that UMAD1 specifically functions on the partially redundant arm of the ESCRT-I and ESCRT-II abscission pathway and that, following knockout of *UMAD1* (or TSG101 knockdown), ALIX compensates for the reduction in the pool of ESCRT-I capable of facilitating abscission. Thus, synergistic abscission delays in HeLa^ΔUMAD1^ cells partially depleted of ALIX are fully consistent with previous studies demonstrating the synergy between TSG101 and ALIX (Christ et al., 2016). The functional redundancy between UMAD1-containing ESCRT-I and ALIX is likely to contribute to the functional plasticity of the cytokinetic machinery, as these two arms might have different contributions in tissue environments with diverse expression of ESCRT-associated factors. Accordingly, recent studies have shown that expression of VPS37B is decreased in advanced colorectal cancer (Kolmus et al., 2021). Alterations in VPS37B-to-VPS37C expression ratios could fine tune ESCRT-I composition, offering a cell type-specific response to differential cytokinetic requirements. Thus, a weaker interaction of UMAD1 with VPS37B could act as a compensatory mechanism in cells with low VPS37C expression levels to ensure the continued formation of cytokinetic ESCRT-I. Compensatory mechanisms between ESCRT-I and ALIX have previously been suggested owing to the adaptation of HIV-1 to use these two factors for efficient replication, perhaps as a mechanisms to overcome functional imbalances between these cellular factors in different tissue environments (Dunger et al., 1991; Fujii et al., 2009).

Strikingly, we show that ESCRT-III recruitment and dynamic exchange are uncoupled in UMAD1-deficient cells (Fig. 6D). UMAD1 might ensure the correct midbody architecture required for ESCRT-III nucleation, perhaps by facilitating a more stable interaction between CEP55 and ESCRT-I. In fact, evidence from *in vitro* experimental systems shows that early-acting ESCRTs, such as the ESCRT-II–CHMP6 complex, increase the nucleation of CHMP4 (Lee et al., 2015) and that ALIX scaffolds CHMP4 polymers (Pires et al., 2009). In addition to its adaptor function, human ESCRT-I forms higher-order helical structures (Flower et al., 2020), and such assemblies might provide nucleating platforms for ESCRT-III polymer formation. In this context, UMAD1 and ALIX might cooperate at the midbody to facilitate the efficient nucleation of ESCRT-III filaments. UMAD1 depletion might also delay midbody accumulation of ESCRT-III monomers above the threshold required for filament nucleation. Finally, these effects might be indirect as UMAD1 and ALIX could promote midbody recruitment of late-acting factors that facilitate the dynamic exchange of ESCRT-III, such as VPS4 (Fig. 6D).

## MATERIALS AND METHODS

### Plasmid construction and mutagenesis

pCR3.1 (Invitrogen) was used for overexpression of HA-, YFP- or Myc-tagged proteins by transient transfection. pCAGGS was modified to encode a GST tag for overexpression of GST-tagged fusion proteins (Martin-Serrano et al., 2003). All proteins were tagged at the N-terminus unless otherwise stated. The bi-cistronic retroviral packaging vector pCMS28, derived from pMigRI (Pear et al., 1998), was modified by Dr Chad Swanson (King's College London, UK) to replace the GFP gene with a puromycin resistance gene linked via an internal ribosome entry site (IRES) to a multiple cloning site containing mCherry- or YFP- followed by the restriction enzymes EcoRI-NotI-XhoI. Puromycin resistance was therefore coupled to mRNA expression of the gene of interest. Further modifications of pCMS28 were preformed to generate pNG72, which contains a geneticin resistance gene in place of the puromycin resistance gene. Both plasmids were a gift from Professor Mike Malim (King's College London, UK).

The coding sequence of UMAD1 was amplified by PCR from HeLa-derived cDNA and inserted into pCR3.1-YFP/HA or pCMS28-YFP plasmids for transient or stable expression of fusion proteins. Mutant forms of HA–UMAD1 were generated using overlap extension PCR. Plasmids based on pCR3.1 that express HA-tagged VPS37 subunits or Myc–VPS28 and pCAG-GST-TSG101 have previously been described (Agromayor et al., 2012).

### Cell lines

HeLa cells were obtained from ATCC (CRM-CCL-2). 293T cells were obtained from ATCC (CRL-3216). All cells were maintained at 37°C and 5% CO_2_ in a humidified atmosphere in Dulbecco’s Modified Eagle Medium (DMEM; Gibco, MA, USA) supplemented with 10% fetal bovine serum (FBS; Sigma-Aldrich, MO, USA) and 20 μg/ml gentamicin (Life Technologies, MA, USA). Cells were regularly tested for the presence of mycoplasma.

### Generation of stable cell lines

Stable cell lines were generated using the MLV-based retroviral packaging vectors pCMS28 and pNG72 encoding the gene of interest as described previously (Ventimiglia et al., 2018). Briefly, 293T producer cells were transfected with 900 ng retroviral packaging construct, 900 ng MLV Gag-pol and 200 ng pHIT-VSV-G using polyethylenimine (PEI; Polysciences, Germany). At 48 h post transfection, viral supernatant was harvested, filtered using 0.22 μm Millex PVDF sterile filters (Millipore) and used to transduce cells treated with 8 μg/ml polybrene (Merck Millipore, Germany). Selection with puromycin (200 ng/ml, Sigma-Aldrich) or G418 (500 μg/ml, Invitrogen, Thermo Fisher Scientific) was applied 48 h later, and cells were passaged under continual selection thereafter. Cell lines stably expressing mCherry–tubulin or CHMP4B–L–GFP have been previously described (Agromayor et al., 2009; Ventimiglia et al., 2018).

### Generation of CRISPR/Cas9 knockout cell clones

Guide RNAs targeting Cas9 endonuclease cleavage upstream (5′) and downstream (3′) of the UMAD1 gene locus respectively were designed using the Integrated DNA Technologies gRNA design tool (https://www.idtdna.com/site/order/designtool/index/CRISPR_CUSTOM) and cloned into lentiCRISPRv2GFP (Sanjana et al., 2014). Sequences are as follows: 5′F, 5′-CACCGTGTCGGCTGACATCTAGAGA-3′; 5′R, 5′-AAACTCTCTAGATGTCAGCCGACAC-3′; 3′F, 5′-CACCGACTGTTTAAGGTCCTCAGCA-3′; and 3′R, 5′-AAACTGCTGAGGACCTTAAACAGTC-3′.

HeLa and 293T cells were transiently transfected with both start and end guide plasmids in one well of a six-well plate. 36 h later, single GFP expressing cells were FACS sorted into wells of 2×96-well plates, and following growth, mRNA was extracted, reverse transcribed to generate cDNA which was used as a template for PCR to detect UMAD1 (Fig. S2).

Primers used to check UMAD1 PCR were as follows: UMAD1checkF, 5′-GTATCAGACCCTGAGATGGAAAATAAGG-3′; and UMAD1checkR, 5′-GGGAGAAAGGAAGTAGAACTTAAG-3′.

### Nucleic acid transfections

293T cells were transfected with plasmid DNA using Polyethylenimine (PEI) (Polysciences), whereas HeLa cells were transfected (when producing CRISPR/Cas9 knockout cells) using Lipofectamine 3000 (Invitrogen) according to the manufacturers' instructions.

Both HeLa and 293T cells were transfected with siRNA using Dharmafect1 (Dharmacon) or Lipofectamine RNAiMAX (Thermo Fisher Scientific). All siRNA transfections were performed in wells of a 24-well plate, using a reverse transfection procedure, according to the manufacturer's instructions. 50 pmol total siRNA per well was generally used unless otherwise stated. Non-targeting siRNA was used to obtain this final concentration in the case of siRNA titration experiments and partial ALIX depletions. siRNA oligonucleotides used were as follows: non-targeting siRNA, ON-TARGETplus Non-targeting (Cat. No. D-001810-01, Dharmacon; siTSG101, 5′-CCUCCAGUCUUCUCUCGUC-3′ (custom order siRNA, Dharmacon); siALIX, 5′-GAAGGAUGCUUUCGAUAAAUU-3′ (custom order siRNA, Dharmacon); and siUMAD1, 5′-GCGAAAACUUAUCACGGUU-3′ (cat. no. N-183515-16-0002, Dharmacon).

### Protein–protein interaction assays

For GST pulldown co-precipitation assays, 293T cells in wells of a six-well plate were co-transfected with 1 µg of pCAGGS-GST/pCR3.1 HA or Myc-based constructs. Medium was changed ≥6 h later, and cells were washed and lysed 48 h post transfection in pre-chilled lysis buffer [50 mM Tris-HCl pH 7.4, 150 mM NaCl, 5 mM EDTA, 5% glycerol and 1% Triton X-100 and a protease inhibitor cocktail (complete mini-EDTA free, Roche)], using 1 ml lysis buffer per well. Lysates were incubated at 4°C for 15 min and clarified by centrifugation (19,000 ***g*** for 10 min). 100 µl supernatants were added to 20 µl 6β Laemmli buffer to achieve a 1× final buffer concentration. Samples were boiled and kept as inputs. 25 µl washed glutathione–Sepharose beads (GE Healthcare Life Sciences) was added to the remaining 900 µl lysates and incubated for 3 h at 4°C. Beads were washed three times with 1 ml wash buffer (50 mM Tris-HCl pH 7.4, 150 mM NaCl, 5 mM EDTA, 5% glycerol and 0.1% Triton X-100), and protein was eluted by boiling each sample in 100 µl 2× Laemmli buffer. Input and pulldown samples were then resolved by SDS-PAGE and analysed by immunoblotting.

For GFP-trap immunoprecipitation assays, lysis and wash buffer compositions were exactly as described above. Cell lines stably expressing each YFP-tagged bait protein were grown to confluency in 15 cm dishes, following which cells were trypsinised and washed three times with PBS. Cells were lysed in 3.6 ml lysis buffer for 15 min at 4°C and sonicated for 10 s using a Branson Sonifier 250 (microtip). Lysates were clarified and input samples were taken and added to Laemmli buffer, as described above. The remaining lysates were incubated for >12 h at 4°C with 35 µl washed GFP-trap^®^ Magnetic Agarose beads (Chromotek). Beads were then washed three times following which protein was eluted from the beads by boiling in 35 µl 2× Laemmli buffer.

In the case of immunoprecipitations for analysis by mass spectrometry, two 15 cm dishes of cells per bait protein were used and volumes of buffers and beads were scaled up accordingly. All solutions were filtered prior to use and all steps except sonication were performed in a laminar flow cabinet.

### Tandem mass tag-based mass spectrometry

For IP-TMT, 30 µl of each GFP-trap immunoprecipitation eluate sample, corresponding to ∼57 µg protein, was run for ∼1 cm into a commercially available 10% polyacrylamide gel, following which staining with Imperial protein stain (Thermo Fisher Scientific) was performed. Background was removed by de-staining, and gel slices containing all the protein in each sample were excised. Reduction of cysteine residues with dithiothreitol and alkylation with iodoacetamide to form stable carbamidomethyl derivatives was then performed. Trypsin digestion was then carried out overnight, followed by isobaric mass tag labelling of each sample with a different TMT tag (Thermo Fisher Scientific). Labelled samples were pooled and desalted using C18 reversed-phase Zip-Tips (Millipore), before being subjected to LC-MS/MS tandem mass spectrometry.

Peptides were resolved by reversed phase chromatography on a C18 column using an Ultimate 3000 NanoLC system (Thermo Fisher Scientific). Eluate was then ionised by electrospray ionisation using an Orbitrap Fusion Lumos (Thermo Fisher Scientific) operating under Xcalibur v4.1. Acquisition using a ‘Synchronous Precursor Selection with MultinotchMS3’ method (SPS) was employed.

Raw mass spectrometry data were processed into peak list files using Proteome Discoverer v2.2 (PD 2.2; Thermo Fisher Scientific). Signal values across samples were normalised to eliminate variation in signal at the level of sample preparation and data acquisition. Data processed this way were then searched using Mascot search algorithm and Sequest search engine embedded in PD 2.2, against the current version of the reviewed SwissProt *Homo sapiens* (Human) database downloaded from UniProt. Data analysis was performed using Perseus (Tyanova et al., 2016). The mass spectrometry proteomics data have been deposited to the ProteomeXchange Consortium, via the PRIDE partner repository (Perez-Riverol et al., 2022), with the dataset identifier PXD043940.

### Western blotting

Samples were denatured in Laemmli buffer before resolving via SDS-PAGE. Proteins were then transferred onto 0.2 μM nitrocellulose membranes (Protran, GE Healthcare) and probed with the indicated primary and secondary antibodies diluted in 1% milk in wash buffer as described above. For HRP-conjugated secondary antibodies, membranes were further treated with Amersham ECL Prime western blotting detection reagent (GE Healthcare). Membranes were visualised using Li-Cor Odyssey Infrared scanner and software.

Custom-made rabbit polyclonal anti-UMAD1 was raised in three rabbits against amino acid residues 6–23 and affinity purified by Lampire Biologicals, and was used at 1:100. Rabbit polyclonal anti-VPS37C (1:1000) and anti-ALIX antibodies (1:1000) were a gift from Prof. Philip Woodman (University of Manchester, UK) and Prof. Wesley Sundquist (University of Utah), respectively. Other primary antibodies were mouse anti-HA (1:1000; Covance, clone HA.11, 16B12), mouse anti-HSP90 (1:1000; Santa Cruz Biotechnology, cat. sc-13119), mouse anti-GFP (1:5000; Roche, clone 7.1/13.1), mouse anti-TSG101 (1:1000; Abcam, clone 4A10), mouse anti-CEP55 (1:500; Abnova), rabbit anti-VPS37A (1:1000; Proteintech cat. 11870-1-AP), rabbit anti-VPS37B (1:1000; Proteintech cat.15613-1-AP) and rabbit anti-RPA3 (1:1000; Proteintech cat. 10692-1-AP). Secondary antibodies included HRP-linked anti-mouse and anti-rabbit IgG antibodies (1:1000; Cell Signalling Technology), goat anti-mouse IgG IRDye^®^-conjugated 680 nm and goat anti-rabbit IgG IRDye^®^-conjugated 800 nm secondary antibodies (1:10,000; LI-COR Biosciences), and donkey anti-mouse IgG (H+L) Alexa Fluor^®^ 594 nm secondary antibody (1:10,000).

Full uncropped blots showing where the bands provided in the figures have been taken from are included in Fig. S5.

### Clonogenic assay

3000 HeLa or HeLaΔUMAD1 cells were seeded and resuspended in wells of a six-well plate. Cell colonies were left to grow for 10 days, following which colonies were fixed and stained with 0.5% (w/v) Crystal Violet (Sigma-Aldrich) in methanol. Imaging of plates was performed using a Chemi-Doc imaging system (Bio-Rad) from which the area occupied by colonies was scored using Fiji (Schindelin et al., 2012), using the square cropping tool to select a constant area of the base of wells for both cell types. This was then converted into an 8-bit greyscale image and the threshold was adjusted to remove background. A threshold value that was suitable for both wells was selected. The remaining colonies in the image were then selected and their area measured.

### Immunofluorescence

Cells were grown on coverslips and fixed for 20 min with methanol at −20°C or 4% paraformaldehyde (PFA) at room temperature, depending on primary antibody used. Cells were then permeabilised with 0.1% Triton X-100 and blocked with 3% BSA before incubation with the appropriate primary antibodies. Cells were finally incubated with Alexa Fluor-conjugated secondary antibodies (1:100) (Thermo Fisher Scientific) and DNA was stained with Hoechst 33258 (Sigma-Aldrich), if necessary. Coverslips were then mounted using Prolong^®^ mountant (Thermo Fisher Scientific). Imaging was performed with an Eclipse Ti-E Inverted CSU-X1 Spinning Disk Confocal (Nikon) equipped with an Ixon3 EM-CCD camera (Andor).

### Multinucleation assay in fixed cells

Cells were plated into wells of a 24-well plate so that two wells (one with a coverslip and one without) were transfected in parallel with a single dose of each siRNA. 50 pmol siRNA was used for non-targeting siRNA (siNT) and siTSG101, whereas 5 pmol was used for siALIX^P^, keeping the total volume of siRNA used per condition at 100 pmol using siNT. At 24 h post transfection, the cells plated on a coverslip were fixed using methanol and stained for tubulin (mouse anti-tubulin; 1:1000; clone DM1A, Sigma-Aldrich), Lamin B1 (rabbit anti-lamin B1; 1:1000; ab16048, Abcam) and Hoechst 33258 (1:10,000), while the cells in the second well were lysed with 1× Laemmli buffer for subsequent immunoblotting to confirm protein depletion. Cells with more than one nucleus were considered multinucleated and dividing cells still connected by midbodies were excluded from scoring. 300 cells per condition were scored. All microscopy analysis was undertaken by a researcher who was not aware of the experimental conditions.

### Live-cell imaging and analysis

For analysis of abscission timings, HeLa^WT^ or HeLa^ΔUMAD1^ stably expressing mCherry–tubulin were transfected with siRNA and plated in wells of a 24-well glass bottom imaging plate (Eppendorf), pre-treated with poly-L-lysine (Sigma) for 20 min at 37°C. Medium was changed 12 h later and 12 fields of view for each condition and three 0.6 µm *Z*-stacks were acquired for 24 h every 10 min. Imaging was performed using a Nikon Ti-Eclipse widefield inverted microscope (Nikon, 40×0.75 N.A. dry objective lens) controlled by NIS-Elements and equipped with a perfect focus system and 37°C microscope chamber (Solent Scientific) supplied with 5% CO_2_. Abscission time was taken as the time interval between midbody appearance to midbody scission.

For UMAD and TSG101 colocalisation studies, HeLa^ΔUMAD1^ cells stably co-expressing YFP–UMAD1 and mCherry–TSG101 were imaged live every 5 min until abscission completion with an Eclipse Ti-E Inverted CSU-X1 Spinning Disk Confocal (Nikon) equipped with an Ixon3 EM-CCD camera (Andor). Before imaging, cells were incubated with silicon-Rhodamine tubulin (SiR–tubulin, Spirochrome, CO, USA) for 1 h at a final concentration of 100 nM. Localisation of YFP–UMAD1 and mCherry–TSG101 was scored after anaphase, upon conversion of the midzone into the midbody and complete furrow ingression.

To establish hierarchy of recruitment for UMAD to the midbody, HeLa^ΔUMAD^ cells stably expressing YFP–UMAD and mCherry–tubulin were depleted of TSG101 or CEP55 and imaged live. Midbodies were scored for the presence of YFP–UMAD by measuring the mean signal intensity within three regions of interest (ROI); one at the centre of the midbody, one in the cytoplasm and one outside the cell as background control. Each cell was given a score by dividing the measures as follows: (mean midbody intensity−mean background intensity)/(mean cytoplasmic intensity−mean background intensity). The same analysis was performed using HeLa^WT^ and HeLa^ΔUMAD^ cells stably expressing YFP–TSG101 and treated with SiR–tubulin.

### Fluorescence recovery after photobleaching experiments

HeLa^WT^ and HeLa^ΔUMAD^ cells expressing CHMP4B–L–GFP were sorted for equal GFP expression. Cells were transfected with siNT or siALIX as described above. Cells were incubated with SiR–tubulin for 3 h (100 nM). Live cells were imaged using a Nikon A1R point-scanning confocal microscope. Midbody-stage cells were identified, from which a stimulation ROI box (used for all midbodies) was placed around CHMP4B–L–GFP at the midbody. Ten pre-bleach frames, one frame at stimulation and 100 post-bleach frames were acquired continuously at 2 s per frame. Stimulation used 90% laser power. Images were analysed using Fiji (Schindelin et al., 2012), where CHMP4B–L–GFP midbody intensities for all timelapses were measured, combined then normalised to the minimum intensity (=0) and maximum intensity (=1) before being pooled for each treatment group and averaged.

### AlphaFold2 prediction of ESCRT-I

Google Colaboratory (Colab) was used to access the AlphaFold2 notebook, which was used for structure predictions (Jumper et al., 2021; Mirdita et al., 2022). The structure of a previously solved human ESCRT-I headpiece containing VPS37B 97–167, TSG101 308–388, VPS28 1–122 and MVB12A 206–228 was predicted and aligned against the experimental structure (PDB: 6VME; white) (Flower et al., 2020) in the PyMOL Molecular Graphics System (Version 2.0 Schrödinger) using the ‘Align’ method and removing outlier rejection to give a root mean square deviation (RMSD) of 2.083 Å across 2093 atoms. Subsequently, the equivalent region of an ESCRT-I headpiece containing VPS37C, TSG101, VPS28 and UMAD1 was predicted (VPS37C 91–161, TSG101 308–388, VPS28 1–122 and UMAD1 78–100).

### Statistical analysis, graphing and figure assembly

Graphs and statistical tests were made using GraphPad Prism 9 (GraphPad, CA, USA). In all cases, error bars are mean±s.e.m. and all experiments are representative of at least three biological replicates. Data were analysed using an unpaired two-tailed Student's *t*-test (for two datasets) or unpaired one-way ANOVA test with Tukey post-hoc multiple comparisons (for multiple datasets). Details of statistical significance and information about sample size are included in their respective figure legends. n.s. (*P*>0.05) represents no significant difference. Statistical difference is shown as *P<0.05, **P<0.01, ***P<0.001 and ****P<0.0001. Movies and images were analysed and assembled using Fiji (Schindelin et al., 2012) and Photoshop (Adobe). All final figures were assembled in Illustrator (Adobe).

## Supplementary Material

10.1242/joces.261097_sup1 Supplementary information
