# Supplementary material for: UMAD1 contributes to ESCRT-III dynamic subunit turnover during cytokinetic abscission
Source: J Cell Sci. 2023 Aug 10;136(15):jcs261097. doi: 10.1242/jcs.261097 (PMC10445733; doi:10.1242/jcs.261097)
Supplement: Supplementary information [file joces-136-261097-s1.pdf]

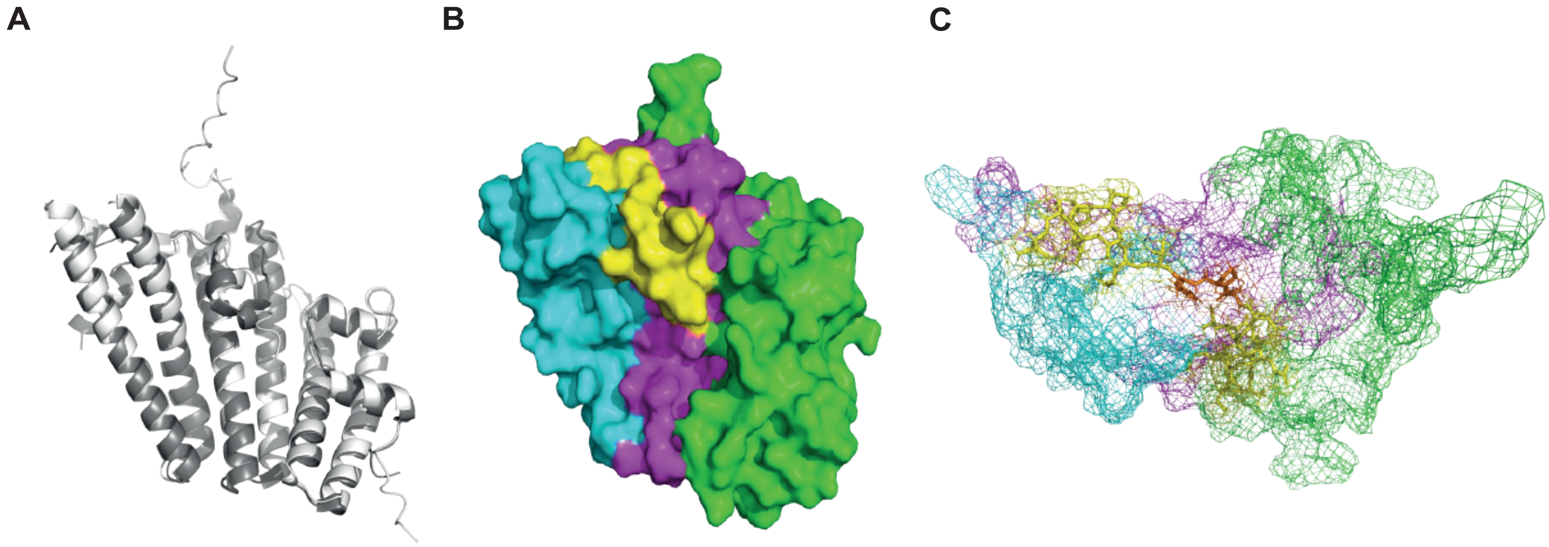

Fig. S1. AlphaFold 2 prediction of ESCRT-I headpiece containing TSG101, VPS28, VPS37C and UMAD1. (A) Alignment of the predicted (grey) and previously solved (white) human ESCRT-I headpiece containing VPS37B (residues 97-167), TSG101 (residues 308-388), VPS28 (residues 1-122) and MVB12A (residues 206- 228) showing a perfect match between the predicted and experimental structures. **(B)** Surface representation of the predicted ESCRT-I headpiece containing VPS37C (residues 91-161, cyan), TSG101 (residues 308-388, magenta), VPS28 (residues 1- 122, green) and UMAD1 (residues 78-100, yellow). **(C)** ESCRT-I headpiece as in (B) represented as a space filled map with lines representing UMAD1 (yellow) and the VPF motif (orange) at the core of the headpiece.

Figure S2.

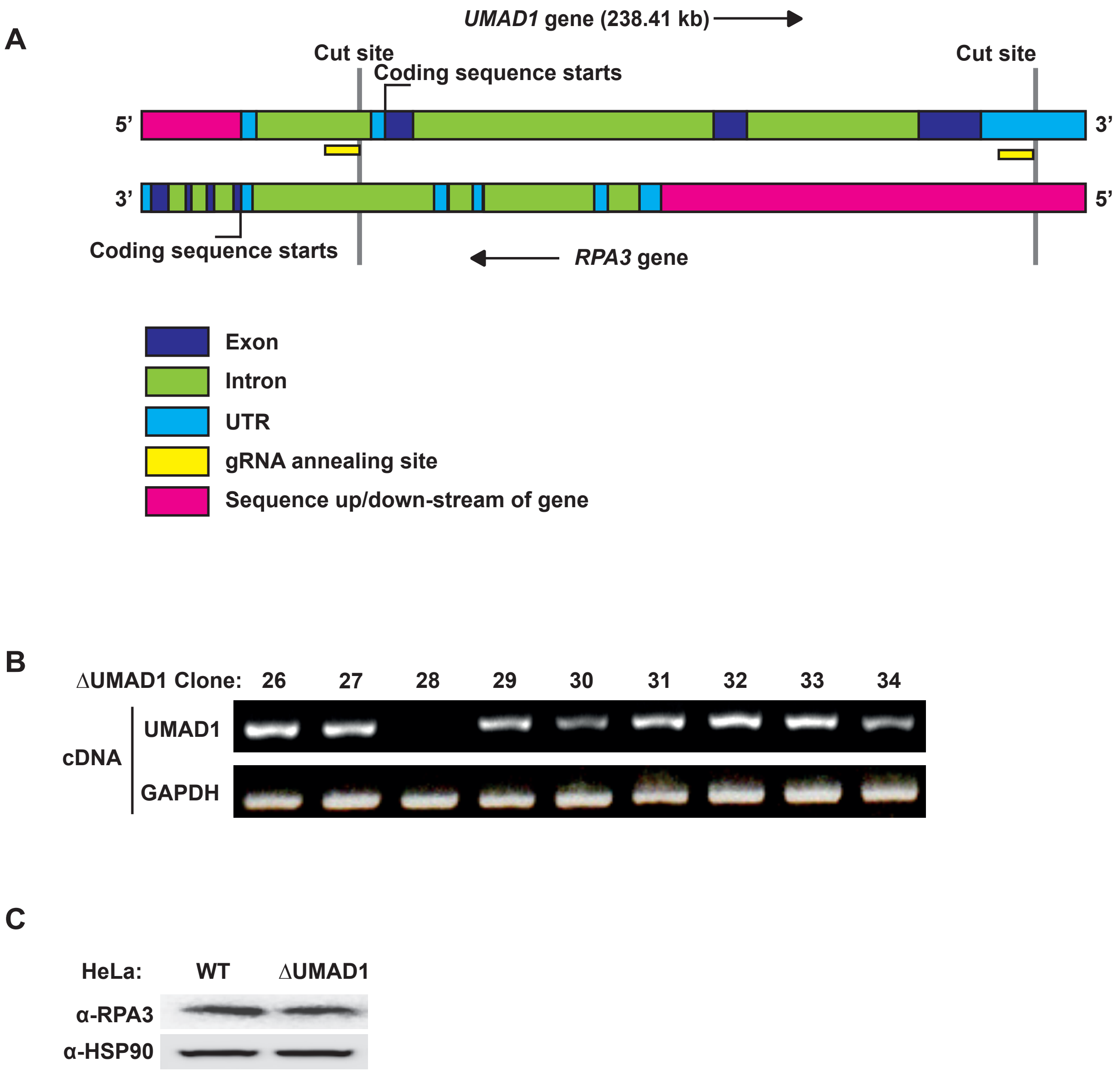

Fig. S2. **Strategy used to generate UMAD1 knockout (ΔUMAD1) cells. (A)** Diagram of UMAD1 gene organisation, including the partially overlapping and oppositely transcribed RPA3 gene. Expected CRISPR/Cas9 cut sites and guide RNAs (gRNA) annealing sites are shown. **(B)** Confirmation of UMAD1 deletion in the selected HeLa$^{\Delta UMAD1}$ clone (clone 28) by PCR of cDNA extracted from cells. GAPDH is used as loading control. **(C)** Side by side comparison of RPA3 expression in HeLa^{WT} and HeLa$^{\Delta UMAD1}$ (clone 28), as shown by western blot, confirming that the RPA3 gene is not disrupted in ΔUMAD1 cells. HSP90 is used as loading control.

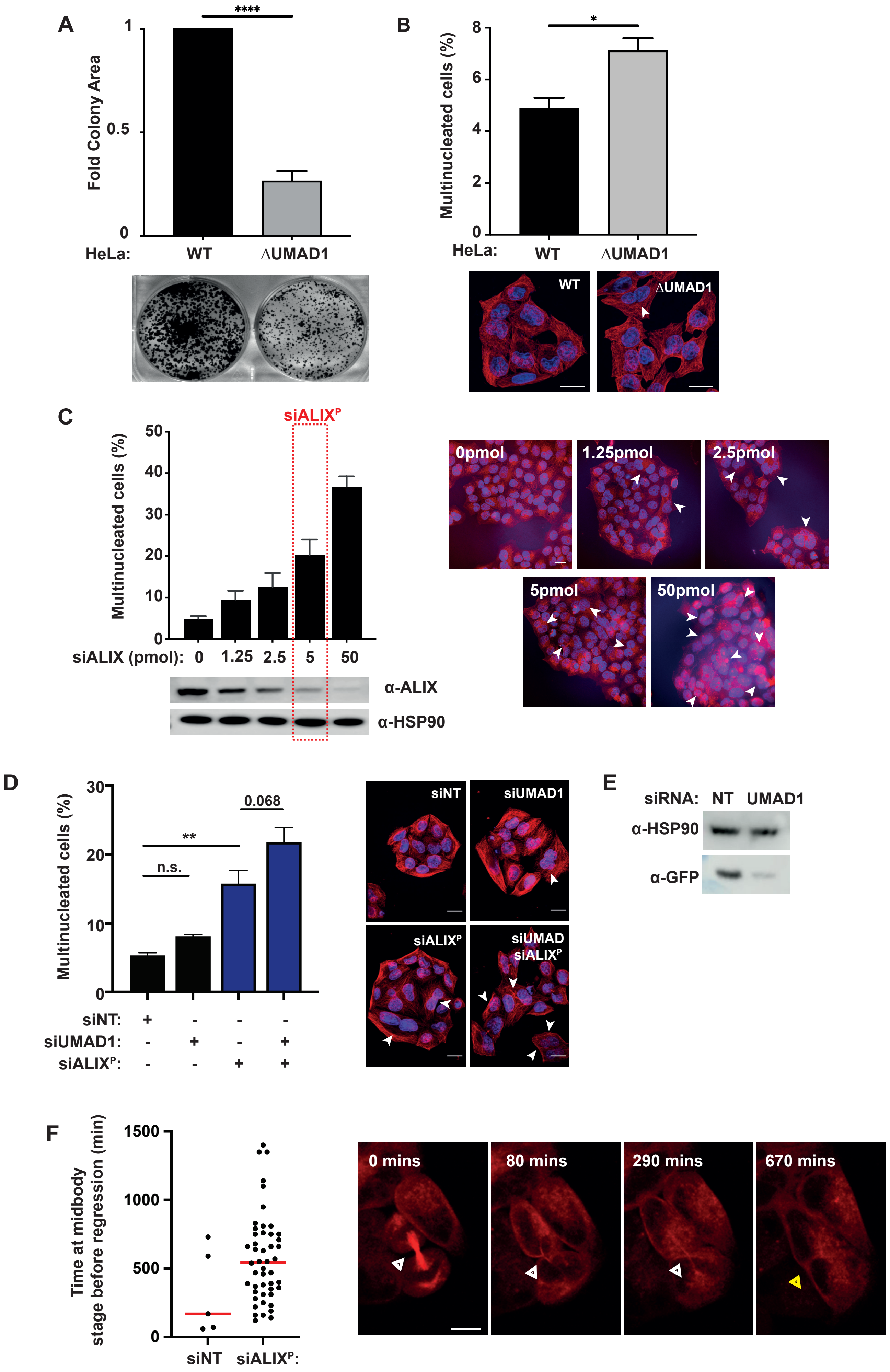

Journal of Cell Science • Supplementary information

Fig. S3. UMAD1 is required for cytokinetic abscission. (A) Clonogenic assay using HeLa^{WT} and HeLa$^{\Delta UMAD1}$ cells. Colony area values are normalised to those of HeLa^{WT} cells. Graph shows the mean ±SEM (n=6). ****$p < 0.0001$ (two-tailed, unpaired Student's t test). Representative image of colony formation is shown below graph. **(B)** Quantification of multinucleation in HeLa^{WT} and HeLa$^{\Delta UMAD1}$ cells. Error bars are mean ±SEM; n≥300 cells from three independent experiments; *$p<0.05$ (two-tailed, unpaired Student's t test). Representative confocal images of cells stained for DNA (blue) and α-tubulin (red) are shown. White arrowheads indicate multinucleated cells. Scale bars, 15μm. (**C**) HeLa cells were treated with increasing amounts of siALIX and scored for multinucleation. Representative confocal images of cells stained for DNA (blue) and α-tubulin (red) are shown. Scale bars, 20μm. ALIX depletion is shown by western blot. HSP90 is used as loading control. 5 pmol (red rectangle) was selected for further experiments, from here onwards siALIX^{P} **(D)** HeLa cells were transfected with the indicated siRNA and scored for multinucleation. Error bars are mean ±SEM; n ≥ 300 cells from three independent experiments; n.s. $p>0.05$ and **$p<0.01$ (unpaired one-way ANOVA test). Representative confocal images shown as above. Scale bars, 15μm. **(E)** 293T cells were co-transfected with a plasmid expressing YFP-UMAD1 and siRNA control (NT) or against UMAD1 (siUMAD1). Cell lysates were collected and used for western blot. Effectiveness of siUMAD1 on YFP-UMAD1 expression was checked with an antibody against GFP. HSP90 was used as loading control. (**F**) Asynchronous cultures of HeLa$^{\Delta UMAD1}$ cells stably expressing mCherry-tubulin were transfected with the indicated siRNA and imaged live. The graph shows the quantification of the time between midbody formation and regression to form a multinucleated cell. A total of 355 cell division events were imaged for siNT, and 165 for siALIX^{P} from two independent experiments, and the multinucleation events from these groups are represented, NT: n=5, siALIX^{P}: n=49 The red line represents the median time. Stills from a representative multinucleation event occurring in cells treated with siALIX^{p} are shown. T=0 represents midbody formation. White arrowheads depict the midbody and the resulting multinucleated cell is indicated with a yellow arrowhead at the time of midbody regression (T=670 min). Scale bar, 10μm. Related to Video S1.

Journal of Cell Science • Supplementary information

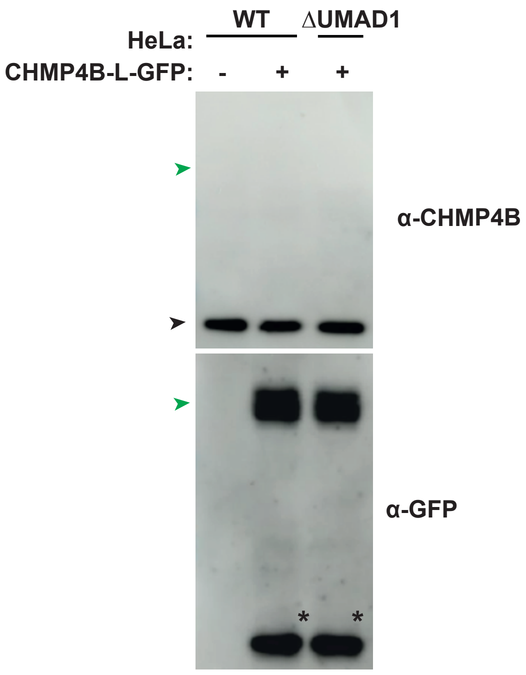

Fig. S4. Sub-physiological expression of CHMP4B-L-GFP. $HeLa^{WT}$ and $HeLa^{\Delta UMAD1}$ cells stably expressing CHMP4B-L-GFP were compared to the parental $HeLa^{WT}$ cells by western blot, using antibodies against CHMP4B or GFP. CHMP4B-L-GFP was undetectable with the anti-CHMP4B antibody. Green arrowheads indicate the expected position of CHMP4B-L-GFP. Black arrowhead indicates the endogenous CHMP4B. Asterisks indicate cleaved GFP products.

Journal of Cell Science • Supplementary information

Figure 1C

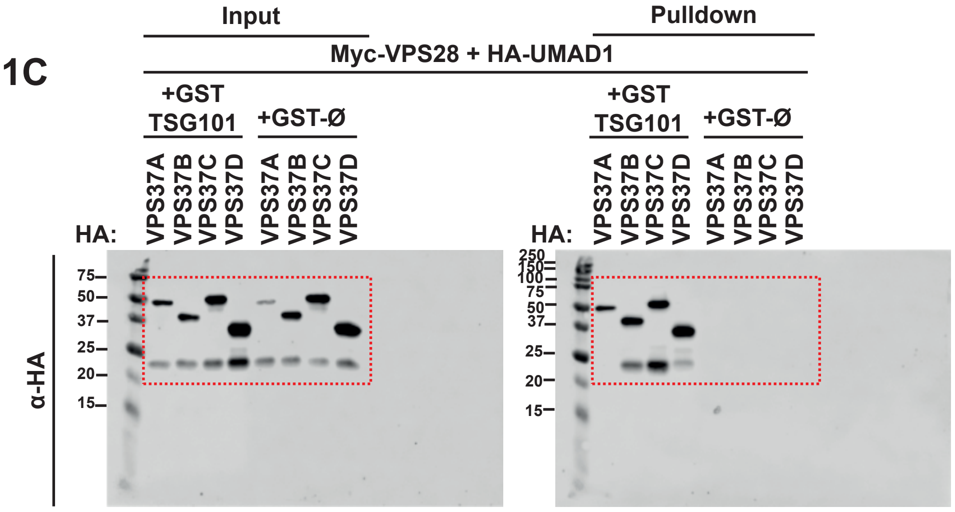

Figure 1D

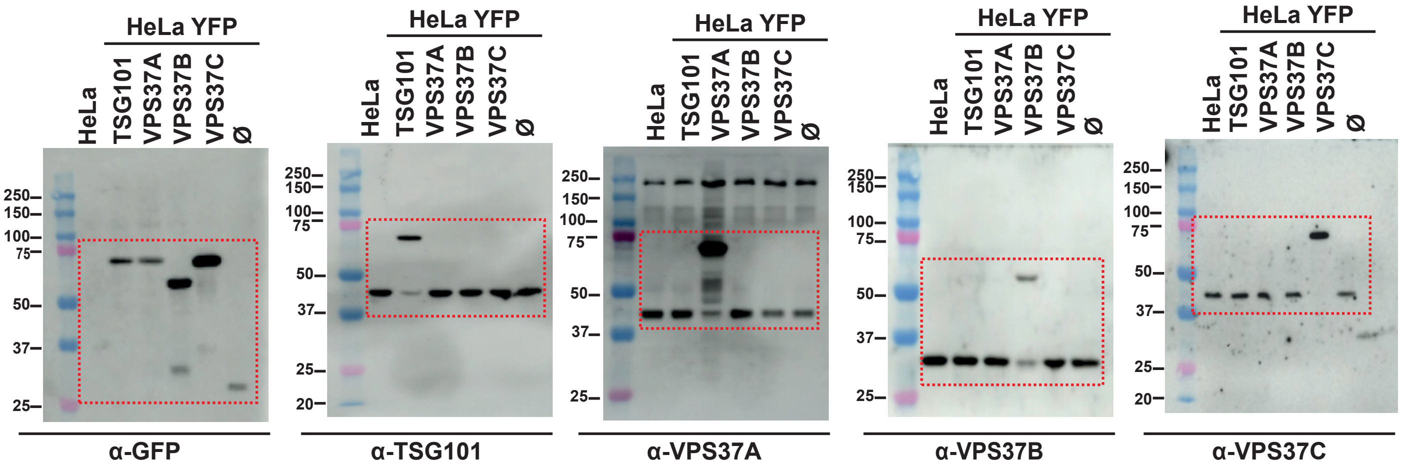

Figure 1E

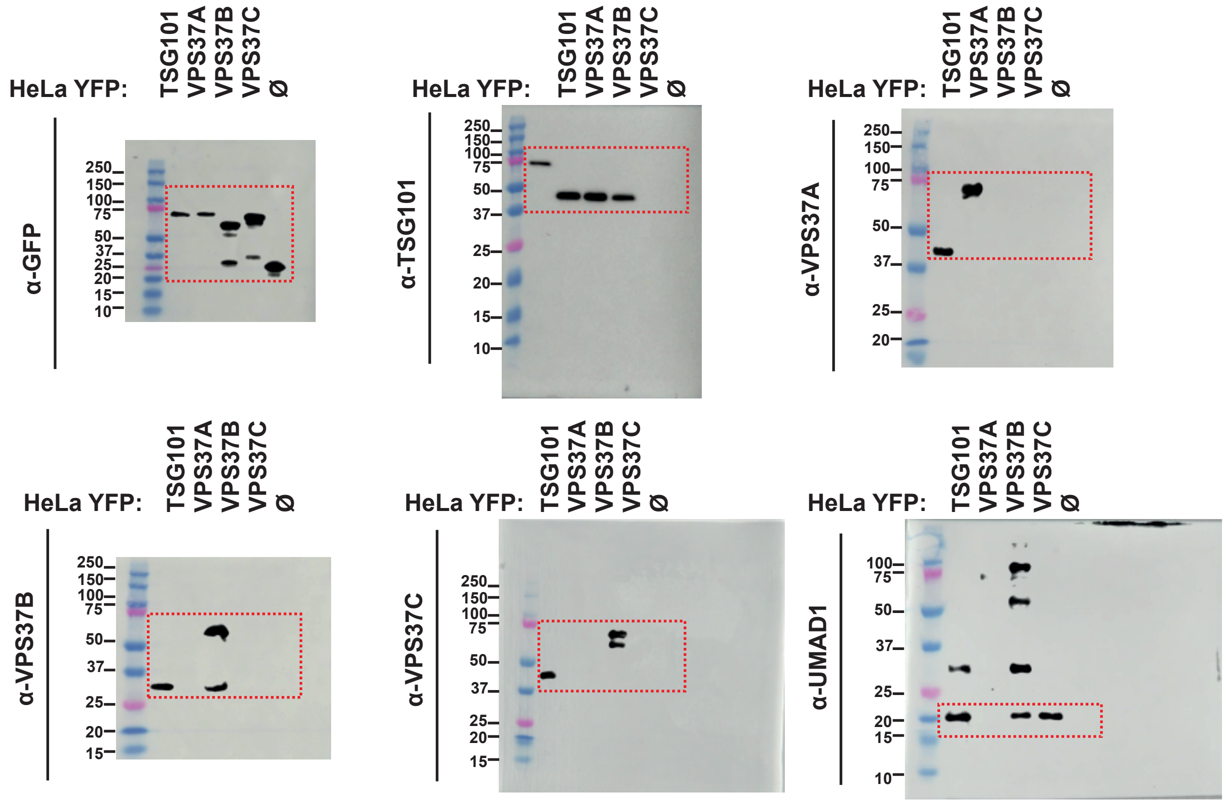

Journal of Cell Science • Supplementary information

Figure 1H

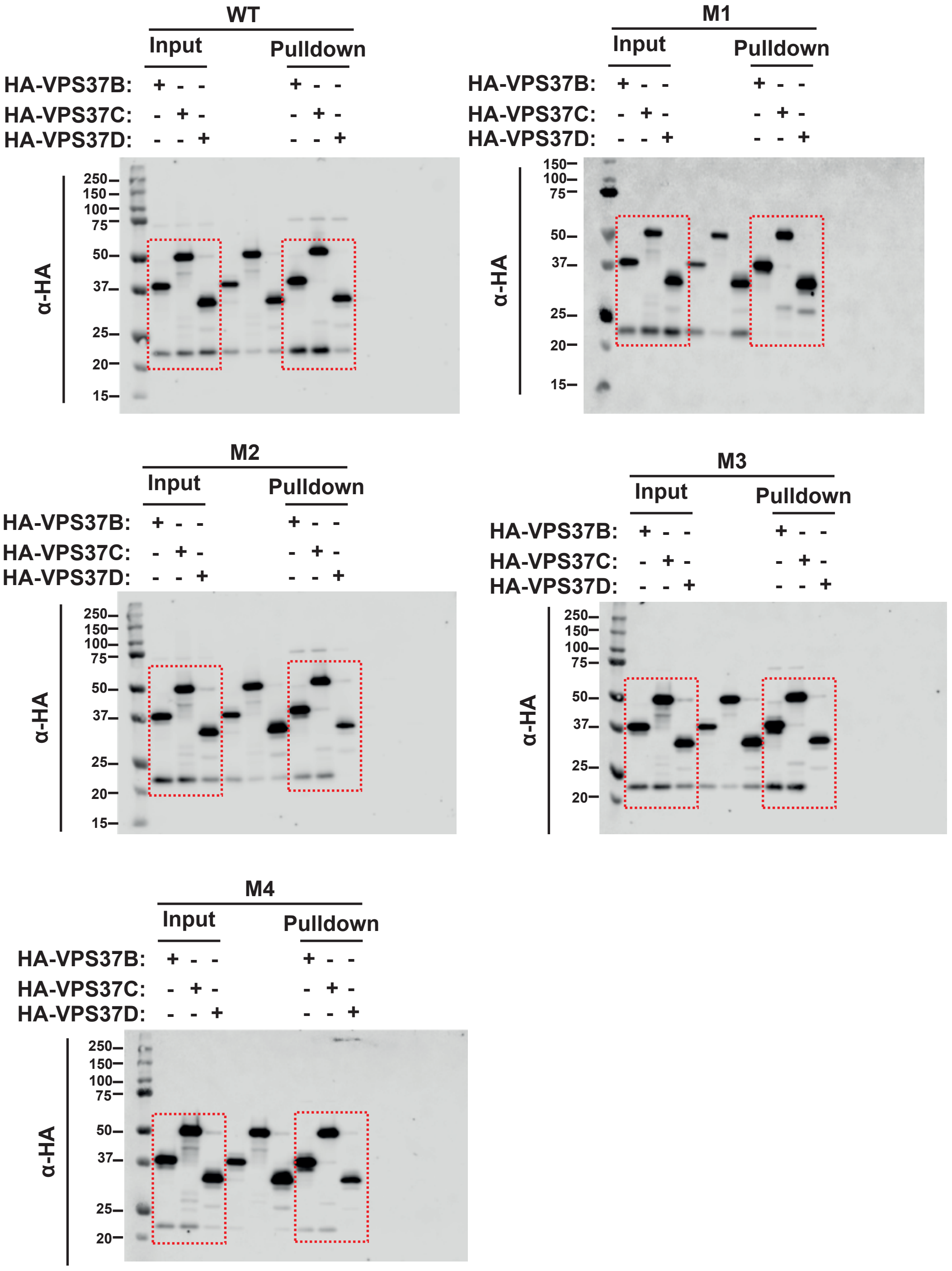

Journal of Cell Science • Supplementary information

Figure 2B

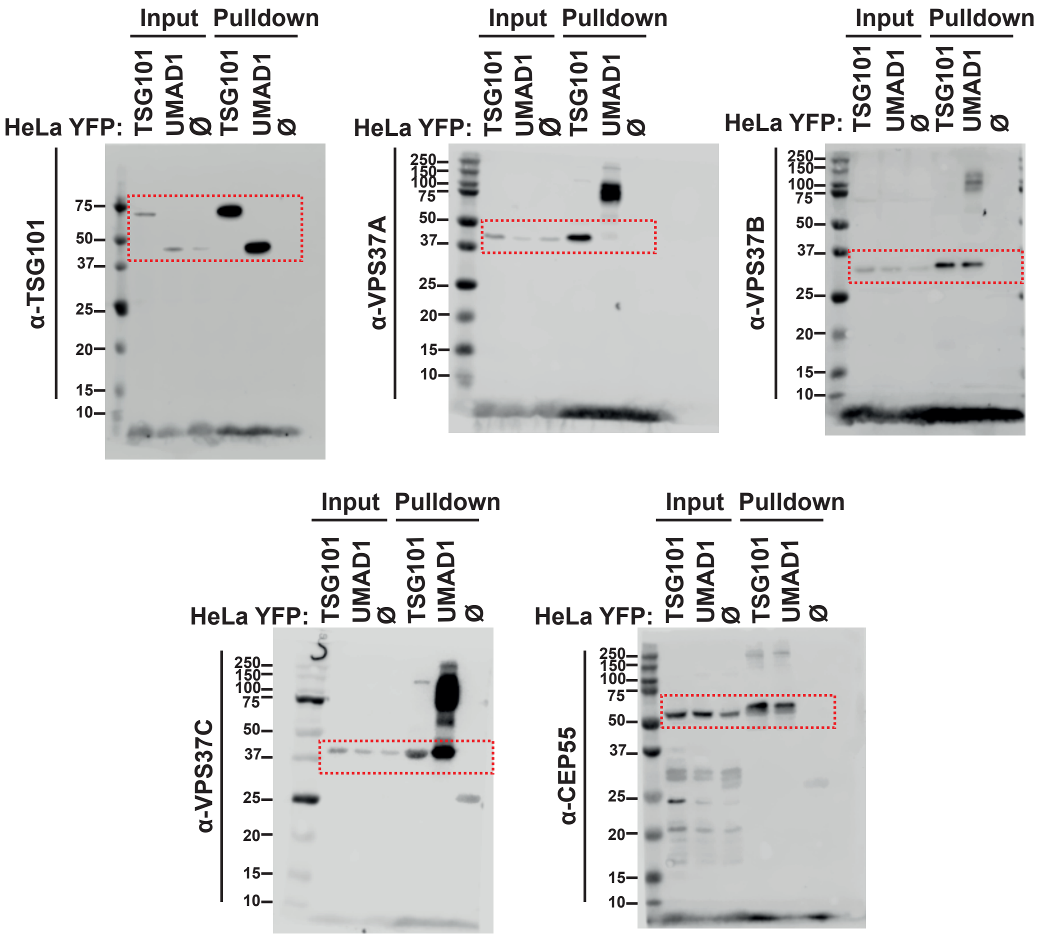

Figure 2D

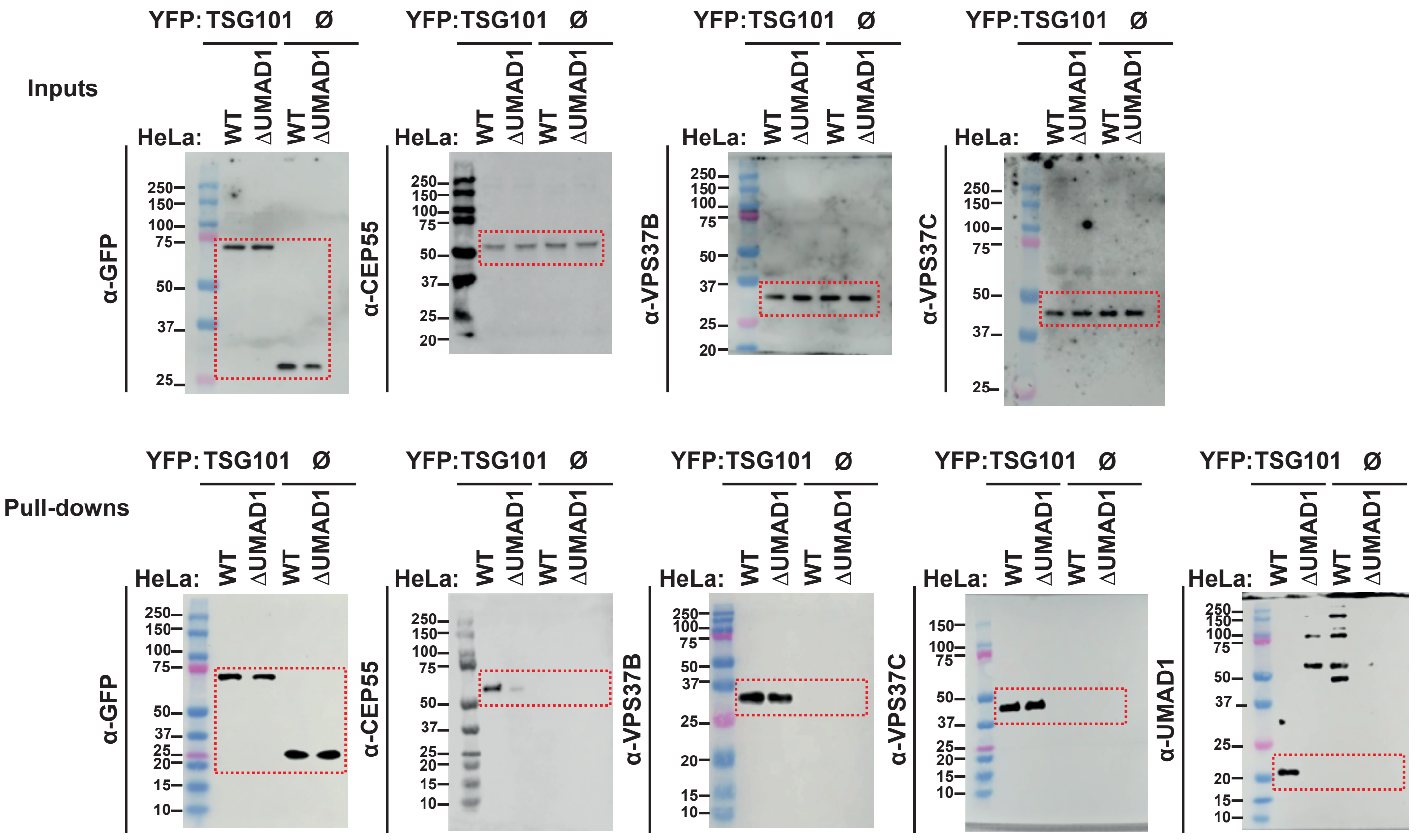

Journal of Cell Science • Supplementary information

Figure 3A

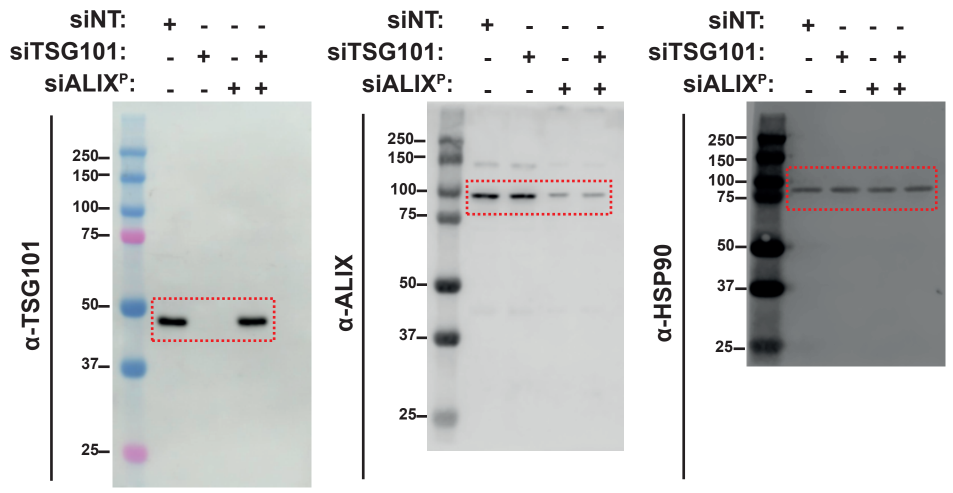

Figure 3B

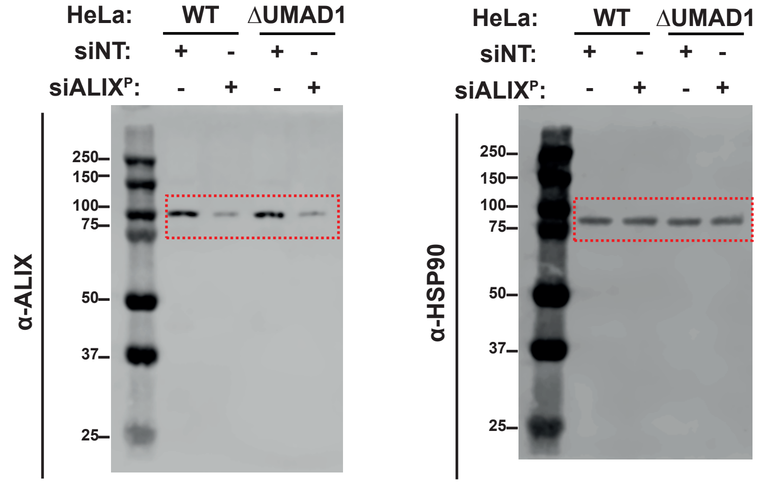

Figure 3C

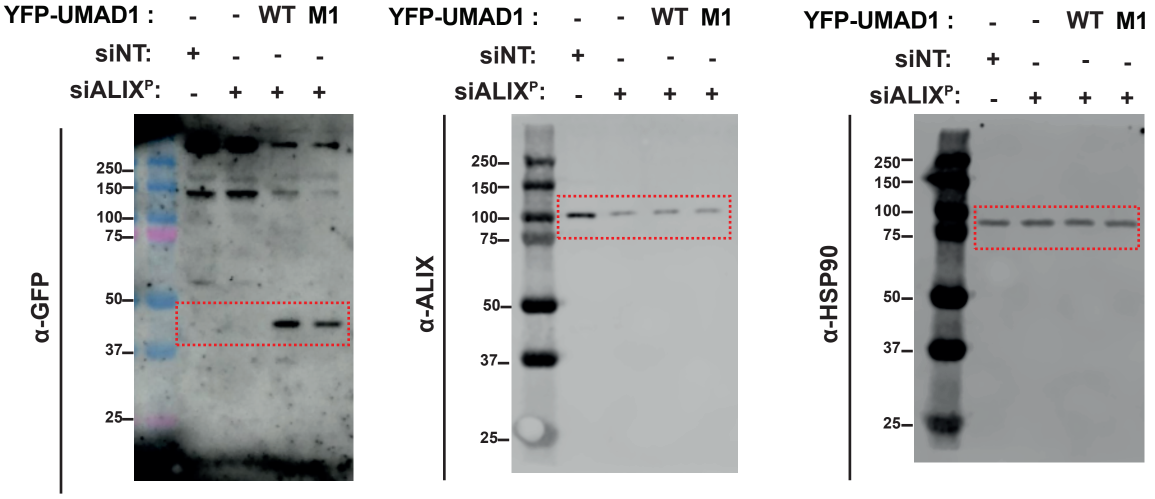

Journal of Cell Science • Supplementary information

Figure 5A

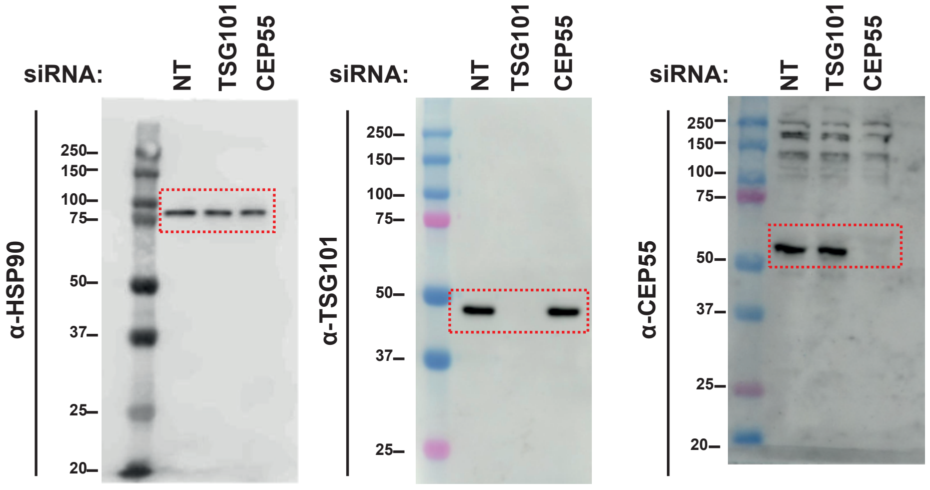

Figure 6A

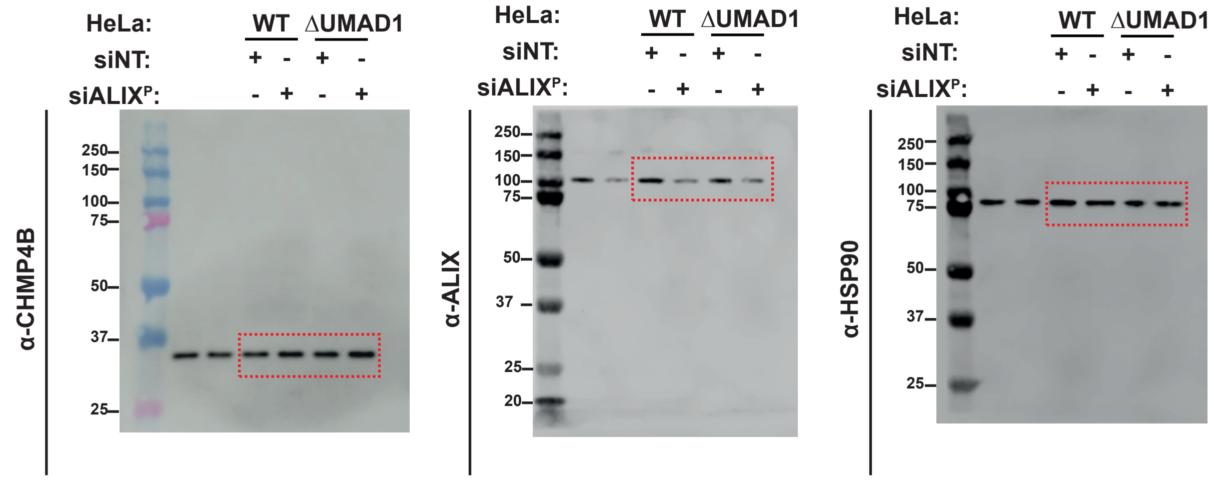

Journal of Cell Science • Supplementary information

Figure S2B

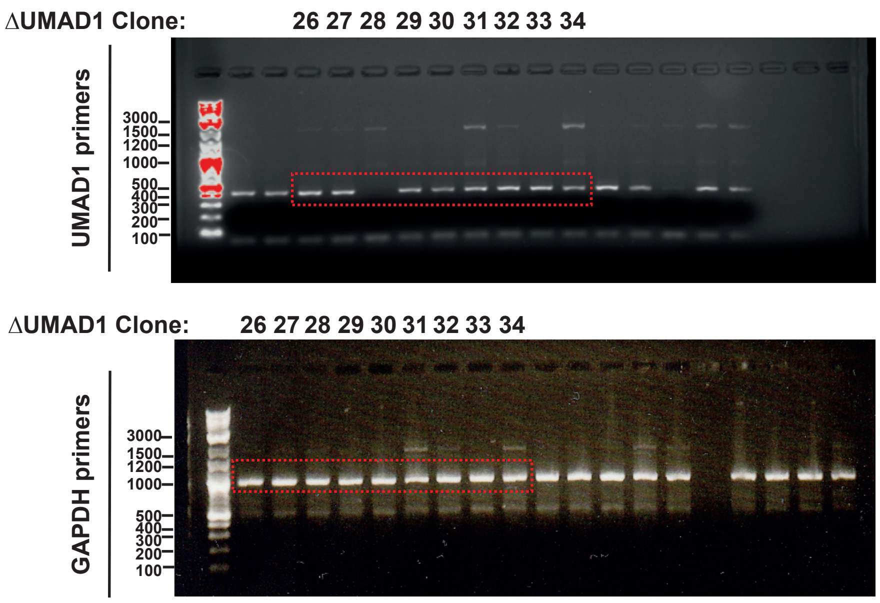

Figure S2C

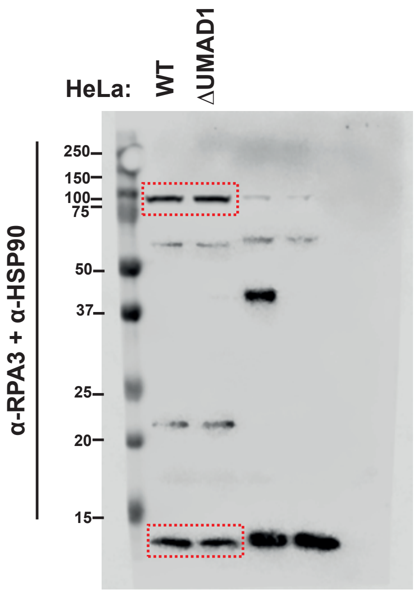

Figure S3A

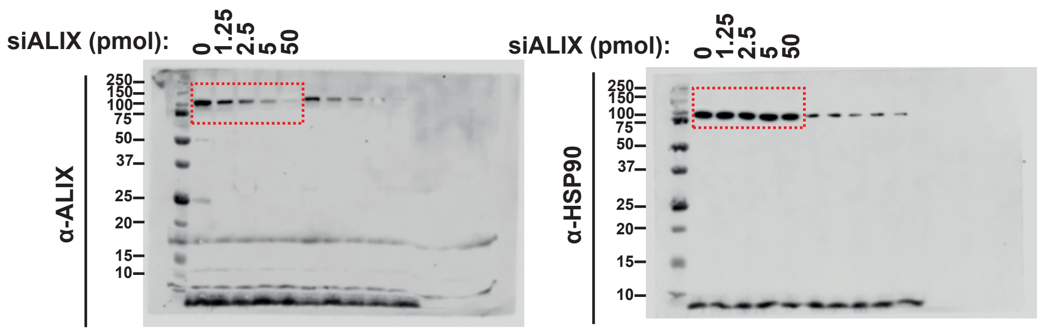

Figure S3C

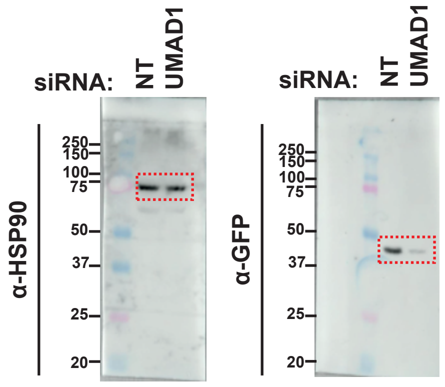

Journal of Cell Science • Supplementary information

Figure S4A

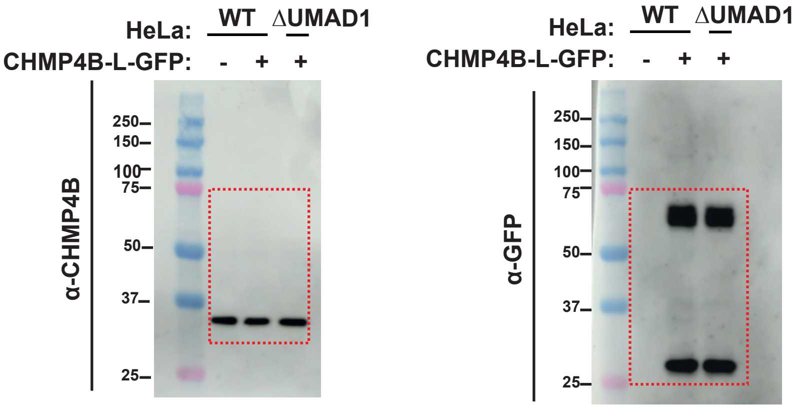

Fig. S5. Blot transparency: uncropped western blots.

Journal of Cell Science • Supplementary information

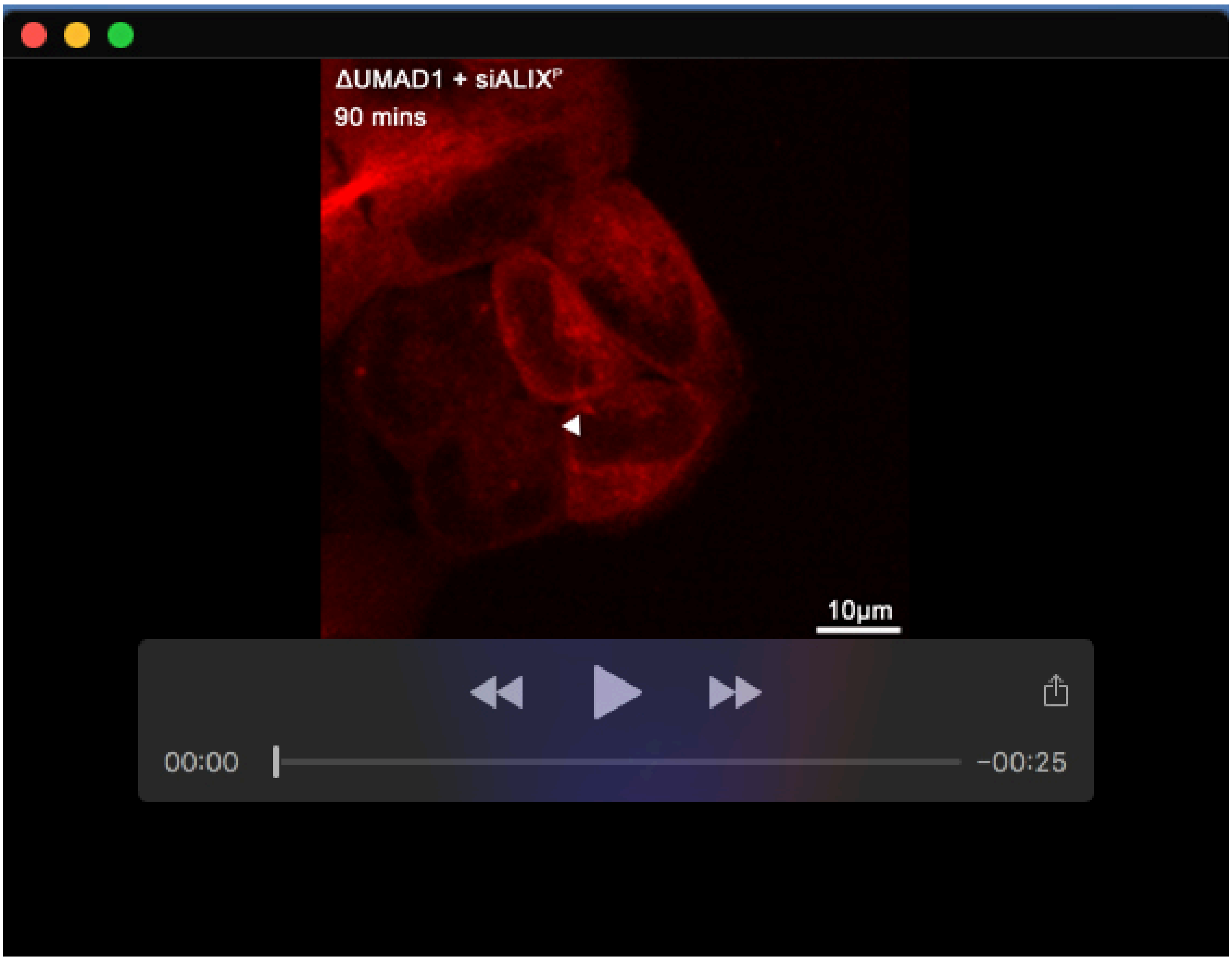

Movie 1. Live imaging microscopy of abscission failure in a HeLa$^{\Delta UMAD1}$ cell stably expressing mCherry-Tubulin following partial depletion of ALIX (siALIX^{P}). Movie was acquired at 1 frame/10 min for 24 hours and played back at 83 frames/8 seconds. Magnification used was 60X. The white arrowhead indicates the midbody and the yellow arrowhead denotes the moment when the two daughter cells coalesce to become a multinucleated cell after being connected by midbody for a prolonged period. Related to Fig. S3F.

Journal of Cell Science • Supplementary information

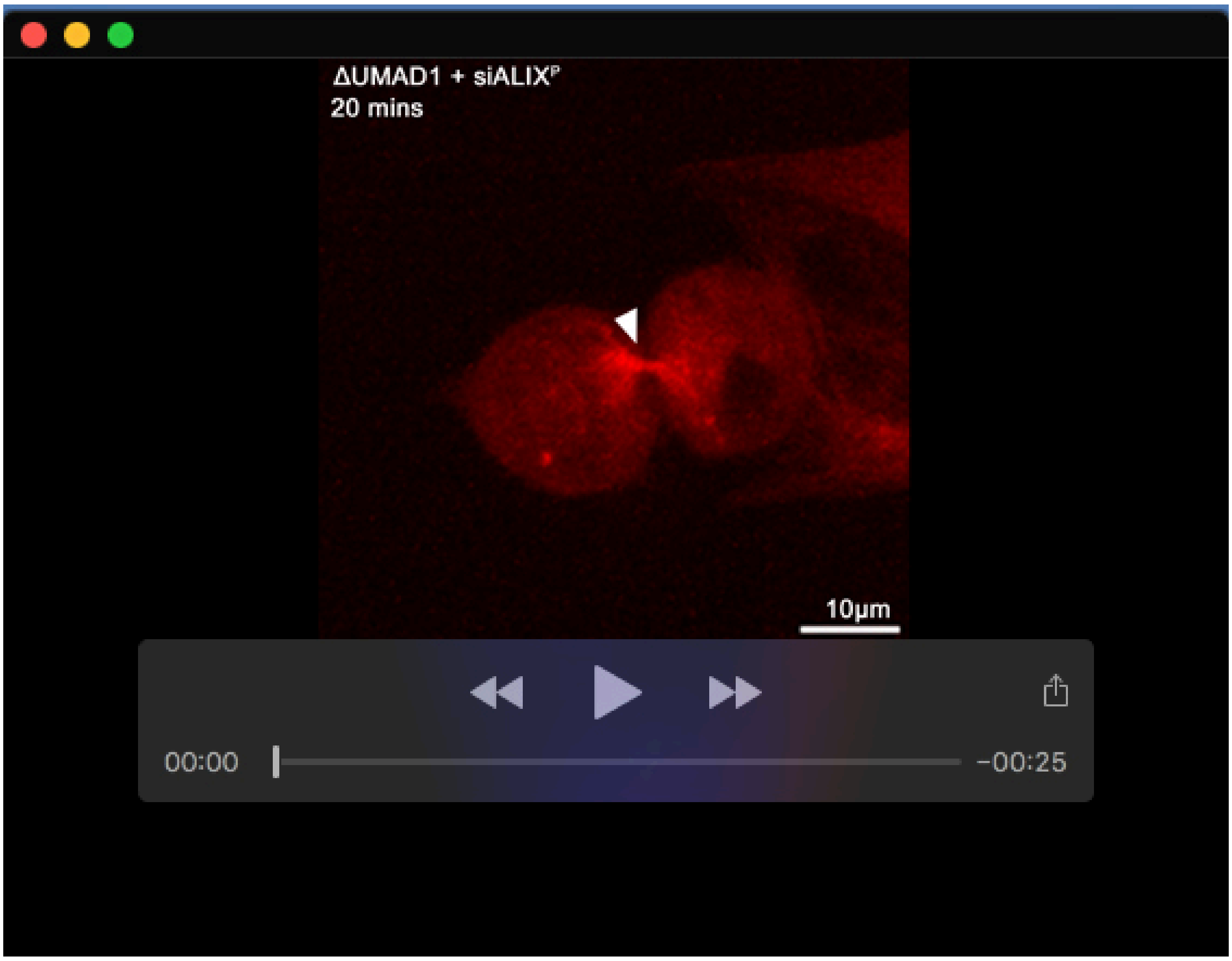

Movie 2. Live imaging microscopy of abscission failure in a HeLa$^{\Delta UMAD1}$ cell stably expressing mCherry-Tubulin following partial depletion of ALIX (siALIX^{P}). Movie was acquired at 1 frame/10 min for 24 hours and played back at 61 frames/12 seconds. Magnification used was 40X. The white arrowhead indicates the midbody and the yellow arrowhead denotes the moment when the two daughter cells coalesce to become a multinucleated cell after being connected by midbody for a prolonged period. Related to Fig. 3C.

Journal of Cell Science • Supplementary information

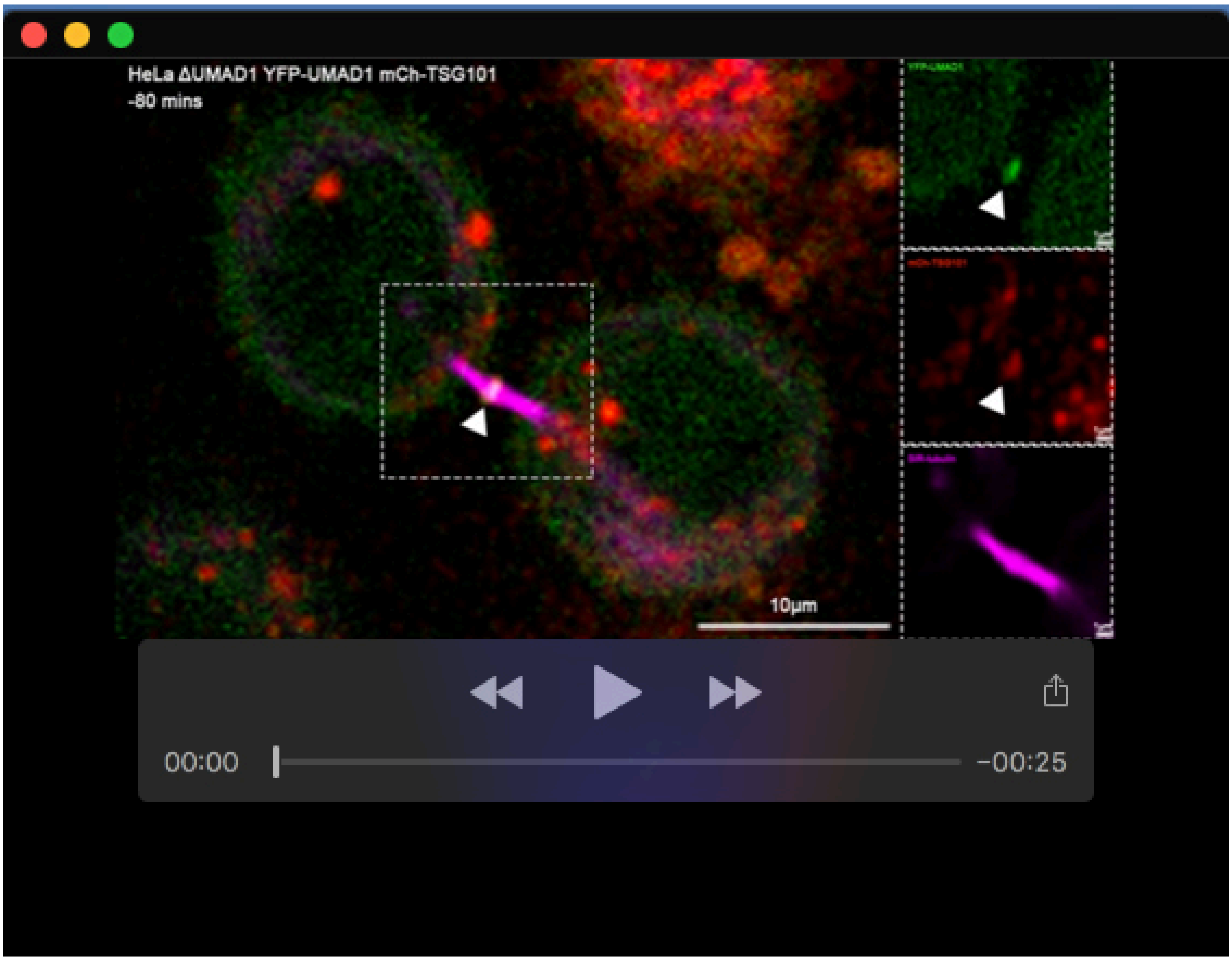

Movie 3. Live imaging microscopy of abscission in HeLa$^{\Delta UMAD1}$ cell stably co-expressing YFP-UMAD1 (yellow) and mCherry-TSG101 (red) and treated with SiR-tubulin (magenta). Movie was acquired at 1 frame/5 min until abscission was observed and played back at 22 frames/8 seconds. Magnification used was 60X. White arrowheads indicate appearance of the fluorescent protein at the midbody. T=0 represents time of abscission. Related to Fig. 4C.

Journal of Cell Science • Supplementary information
