# Supplementary material for: UMAD1 contributes to ESCRT-III dynamic subunit turnover during cytokinetic abscission
Source: J Cell Sci. 2023 Aug 10;136(15):jcs261097. doi: 10.1242/jcs.261097 (PMC10445733; doi:10.1242/jcs.261097)
Supplement: Supplementary file 1 [file joces-136-261097_review_history.pdf]

UMAD1 contributes to ESCRT-III dynamic subunit turnover during cytokinetic abscission

James Glover, Edward J. Scourfield, Leandro N. Ventimiglia, Xiaoping Yang, Steven Lynham, Monica Agromayor and Juan Martin-Serrano
DOI: 10.1242/jcs.261097

Editor: David Stephens

Review timeline
Original submission: 19 February 2023
Editorial decision: 22 March 2023
First revision received: 25 May 2023
Accepted: 26 June 2023

Original submission

First decision letter

MS ID#: JOCES/2023/261097

MS TITLE: UMAD1 determines ESCRT-III dynamic subunit turnover during cytokinetic abscission

AUTHORS: James Glover, Edward J Scourfield, Leandro N Ventimiglia, Xiaoping Yang, Steven Lynham, Monica Agromayor, and Juan Martin-Serrano
ARTICLE TYPE: Research Article

We have now reached a decision on the above manuscript.

To see the reviewers' reports and a copy of this decision letter, please go to: https://submit-jcs.biologists.org and click on the 'Manuscripts with Decisions' queue in the Author Area. (Corresponding author only has access to reviews.)

As you will see, the reviewers are strongly supportive of publication but do raise a number of criticisms that prevent me from accepting the paper at this stage. They suggest, however, that a revised version might prove acceptable, if you can address their concerns. If you think that you can deal satisfactorily with the criticisms on revision, I would be pleased to see a revised manuscript. We may then return it to the reviewers.

I consider that the points raised are all reasonable and should require only amendments to the text and figures. In particular, please do pay attention to the comments relating to how well the data support the conclusions and to the statistical analysis.

Please ensure that you clearly highlight all changes made in the revised manuscript. Please avoid using 'Tracked changes' in Word files as these are lost in PDF conversion.

I should be grateful if you would also provide a point-by-point response detailing how you have dealt with the points raised by the reviewers in the 'Response to Reviewers' box. Please attend to all of the reviewers' comments. If you do not agree with any of their criticisms or suggestions please explain clearly why this is so.

© 2023. Published by The Company of Biologists under the terms of the Creative Commons Attribution License (https://creativecommons.org/licenses/by/4.0/).

Reviewer 1

Advance summary and potential significance to field

This paper reports the identification of a new member of ESCRT-I, provides a complete biochemical dissection of its selective incorporation into a subset of ESCRT-I that also contains VPS37C, and provides convincing functional data for the involvement of UMAD1 in cytokinesis, alongside Alix. The involvement of UMAD1 in regulating ESCRT-III assembly dynamics is particularly intriguing and points to a novel role for upstream ESCRTs in promoting the correct assembly of a functional ESCRT-III polymer. Altogether, these findings will be of broad interest to cell biologists. Indeed, I would consider this paper to be high priority for JCS, subject to some relatively minor improvements in the data, which are somewhat untidy in places.

Comments for the author

In the first paragraph of the results section, it states that the YFP constructs were overexpressed at near endogenous levels. However, these data are not shown. Could the data be included in supplementary information please? (Or are these cells also used for the data in Fig 1D, in which case this should be referred to).
In Figure 1A the ion ratio of UMAD1 for YFP-TSG101/YFP is quite low for this representative experiment. It would be helpful to know the average ratio over several experiments of UMAD1 for TSG101 and VPS37C, since this is the focus of the paper. It would also be interesting to know if VPS37D was detected in these experiments.
Figure 1C. Can this be quantified? The amount of UMAD1 expressed is much greater in combination with VPS37D in this experiment. It would be nice to get some firmer numbers to properly define the affinity of UMAD1 for VPS37D. Is this due to loading/uneven transfection or a stabilisation? The bars need to be aligned.
Figure 1D. Ideally the panels should also include non-transfected HeLa cells.
Figure 1E. These figure panels look very bleached out and over-contrasted. Could the contrast be amended to show the background please? Please asterisk the additional low MW GFP bands in the VPS37B/C lanes and explain in the text or legend – presumably cleaved YFP? As for Figure 1D, inclusion of non-transfected cells as a control would be helpful.
Figure 1H. The panels are cropped very tightly at the bottom, below UMAD1. Quantitation across several replicates would be preferred.
Figure2B. CEP55 appears to have shifted Mwt in the pulldown – can this be noted/explained?
Figure 2D. These are pulldowns but the inputs are not shown. The TSG101 and VPS37B panels are very highly contrasted.
Figure S3E. Could the blot for UMAD1 be shown please?
Figure 3D. It would be helpful to see statistical analysis of these data.
Figure 4. Is it possible to visualise endogenous UMAD1 at the mid-body? How does the timing of UMAD1/TSG101 compare with CEP55?
Figure 5. The description of Figure 5A in the text is somewhat confusing. “Demonstrating that UMAD1 acts downstream (of?) CEP55 and ESCRT”? If UMAD1 is an ESCRT1 component then UMAD1 acts alongside rather than downstream of TSG101. This section could be rephrased. The TSG101 blot is poor quality and very tightly cropped. The panel labelling in the figures (Merge and YFP only) is a little confusing and it would be helpful to reference tubulin in the label. The merges are inconsistent (R-G; M-G) and magenta-green is preferable.
Figure 6A. It would be helpful to see a blot of the CHMP4 in these experiments, to show whether CHMP4B levels are affected by the conditions but also to confirm that CHMP4B-GFP levels are close to endogenous CHMP4B levels. The IF panels need to be labelled, and green-magenta is preferable.
Figure 6B. Can statistical analysis be performed please?

Reviewer 2

Advance summary and potential significance to field

Review UMAD1, Glover, JCS

© 2023. Published by The Company of Biologists under the terms of the Creative Commons Attribution License (https://creativecommons.org/licenses/by/4.0/).

In the submitted manuscript the authors identify UMAD1 as a a new ESCRT-I component and characterize its role in cytokinetic abscission. Moreover, they provide evidence to suggest that UMAD1 is an abscission specific ESCRT-I component. While ESCRTs are known to drive numerous processes in cells, the basis for their specific recruitment to the site of action is still largely unknown. Additionally, while the ESCRT-III and VPS4 ESCRT subcomplexes have been extensively studied in cells our knowledge on the function and regulation of the early ESCRT subcomplexes (ESCRT-I and II) inside cells is limited. Therefore, the findings presented in the manuscript advance our understanding of ESCRTs, in general, and of ESCRT-mediated cytokinetic abscission, in particular. The manuscript is concise, well written and most conclusions are fully supported by the experimental results. I do have several comments which should be addressed prior to publication.

Comments for the author

Comments:
1. My main concern in the manuscript is the FRAP experiments and their interpretation. First, on the technical side, it is not clear to me why the CHMP4B recovery is less than 0.5 (in the Mierzwa 2017 publication the reported recovery is close to 1). This difference should be explained. Second, the results obtained in the delta UMAD1 compared to siRNA Alix, suggests that most of the phenotype is contributed by depleting Alix rather than by depleting UMAD. I understand that depleting both proteins is essential for getting an abscission phenotype due to compensation between the two pathways, but I feel that concluding from these data a connection between UMAD and protein exchange in the ESCRT-III filament is a stretch. I recommend toning down the title and main text in relevant places modifying the model to avoid inferring a direct effect and explicitly refer to this issue in the discussion. Alternatively, more experiments should be performed to substantiate the connection between UMAD and CHMP4B exchange.
2. The authors nicely mapped the interactions with proteins within the ESCRT-I subcomplex and with CEP55, which is an upstream component that recruits ESCRT-I. What about interactions with proteins of the downstream ESCRT-II subcomplex? It will be good to provide experimental data on that or at least to discuss ESCRT-II interactions somewhere in the text.

Minor comments:
1. Fig. 2 – It will be nice to add a scheme summarizing the interactions observed for UMAD1. It is a bit hard to follow.
2. Fig. 2D – grey levels in anti-GFP and anti-VPS37C gels appear too narrow.
Bands are missing the typical haze surrounding the bands as seen in the other panels and throughout the figure. Please replace with less filtered versions.
3. Fig. 3 A-B – Protein loading controls point to a difference in total protein levels. It should not affect the conclusions, but it is important to indicate whether this was taken into account when calculating the percentage of expressed proteins in the different conditions and it should be noted in figure legends. Alternatively, the gels can be replaced with gels in which the total protein levels are similar to avoid confusion.
4. Fig. 3 C – Image quality is poor. Please improve to make sure the intercellular bridge can be clearly seen. Also, what does the yellow arrow indicate? Arrows are not specified in Fig. legend.
5. Fig 3 D – It should be clearly indicated in the cumulative plot that only cells that successfully completed abscission were quantified. It is mentioned in the Fig. legend but given the multinucleated cells phenotype I think it is important to specifically indicate that on the Y axis of the plot.
6. I think it is better to show STDEV rather than SEM.

First revision

Author response to reviewers' comments

© 2023. Published by The Company of Biologists under the terms of the Creative Commons Attribution License (https://creativecommons.org/licenses/by/4.0/).

KING'S College LONDON

University of London

May 25th, 2023

Dear Dr Stephens,

Please find attached the revised version of our manuscript entitled "UMAD1 contributes to ESCRT-III dynamic subunit turnover during cytokinetic abscission", MS ID#: JOCES/2023/261097. We are confident that the constructive comments by the reviewers have been addressed, and the clarity of the manuscript has been improved. A point-by-point response to these comments is included in this letter. We have also formatted the manuscript documents to follow the format guidelines from Journal of Cell Science.

Yours faithfully,

Juan Martin-Serrano
Monica Agromayor

Reviewer 1 Advance Summary and Potential Significance to Field:
This paper reports the identification of a new member of ESCRT-I, provides a complete biochemical dissection of its selective incorporation into a subset of ESCRT-I that also contains VPS37C, and provides convincing functional data for the involvement of UMAD1 in cytokinesis, alongside Alix. The involvement of UMAD1 in regulating ESCRT-III assembly dynamics is particularly intriguing and points to a novel role for upstream ESCRTs in promoting the correct assembly of a functional ESCRT-III polymer. Altogether, these findings will be of broad interest to cell biologists. Indeed, I would consider this paper to be high priority for JCS, subject to some relatively minor improvements in the data, which are somewhat untidy in places.
We thank the reviewer for the unambiguous support of our manuscript and for the constructive comments. We are confident that the minor points have been addressed in the revised manuscript.

Reviewer 1 Comments for the Author:
In the first paragraph of the results section, it states that the YFP constructs were overexpressed at near endogenous levels. However, these data are not shown. Could the data be included in supplementary information please? (Or are these cells also used for the data in Fig 1D, in which case this should be referred to).
This data is shown in Figure 1D and we already reference this in the text. New blots are included to show the parental cells as requested.

In Figure 1A the ion ratio of UMAD1 for YFP-TSG101/YFP is quite low for this representative experiment. It would be helpful to know the average ratio over several experiments of UMAD1 for TSG101 and VPS37C, since this is the focus of the paper. It would also be interesting to know if VPS37D was detected in these experiments.
To increase the clarity in this figure the normalised ratios are converted to $\log_2$. The actual ratio of UMAD1 for YFP-TSG101/YFP is 3.14, which is a clear enrichment over the non-specific background. The data in Figure 2C corresponds to a second independent experiment, and the UMAD1 ratio for YFP-TSG101/YFP is 3, thus there is good consistency in this ratio between the two experiments. To avoid any confusion for the readers, we have now modified the labelling of Figures 1A and 2A to indicate the $\log_2$ conversion.
We have not detected VPS37D by mass spec in these experiments, suggesting that this subunit is not well expressed in Hela cells.

Figure 1C. Can this be quantified? The amount of UMAD1 expressed is much greater in combination with VPS37D in this experiment. It would be nice to get some firmer numbers to properly define the affinity of UMAD1 for VPS37D. Is this due to loading/uneven transfection or a stabilisation? The bars need to be aligned.

© 2023. Published by The Company of Biologists under the terms of the Creative Commons Attribution License (https://creativecommons.org/licenses/by/4.0/).

We have quantified the UMAD1 band intensity in the pull-down and divided this value by the UMAD1 band intensity in the corresponding input samples. These numbers are now included as “UMAD1 binding” in Figure 1C, and a brief description of the quantification is included in the figure legend. We do not make any claim regarding the relative affinity of these interactions as this type of pull down is not an appropriate technique to measure binding affinities.
The bars are now aligned.

Figure 1D. Ideally the panels should also include non-transfected HeLa cells.
This figure now includes a new set of blots showing the non-transduced parental cells.

Figure 1E. These figure panels look very bleached out and over-contrasted. Could the contrast be amended to show the background please? Please asterisk the additional low MW GFP bands in the VPS37B/C lanes and explain in the text or legend – presumably cleaved YFP? As for Figure 1D, inclusion of non-transfected cells as a control would be helpful.
The blots in these panels were unmodified but the signal is very strong, giving it the appearance of over-contrasted. We have now adjusted the brightness to improve clarity and we are providing all the uncropped blots in the supplement. The asterisks marking cleaved YFP-fusions are now included in the figure and explained in the legend.

Figure 1H. The panels are cropped very tightly at the bottom, below UMAD1. Quantitation across several replicates would be preferred.
The panels in this figure have been re-cropped, and the quantification of “UMAD1 binding” is shown as described above.

Figure 2B. CEP55 appears to have shifted Mwt in the pulldown – can this be noted/explained?
The reviewer is right and a subset of CEP55 runs at higher molecular weight in the pull-down fraction. It has been shown in the literature that CEP55 is regulated by phosphorylation (PMID: 21079244). We therefore speculate that UMAD1 may bind preferentially to the phosphorylated form of CEP55. This point is now mentioned in the results section.

Figure 2D. These are pulldowns but the inputs are not shown. The TSG101 and VPS37B panels are very highly contrasted.
New blots containing the inputs are now included in this figure. The UMAD1 blot in the input is not included as the antibody only works in enriched samples. The brightness of the pull-down blots has been adjusted as requested and we are providing all the uncropped blots in the supplement.

Figure S3E. Could the blot for UMAD1 be shown please?
As explained above, showing this blot is not feasible as our antibody against UMAD1 only works in enriched samples such as the pull-downs shown in Figures 1E and 2D.

Figure 3D. It would be helpful to see statistical analysis of these data.
The statistical analysis of these data is now included in the figure legend.

Figure 4. Is it possible to visualise endogenous UMAD1 at the mid-body? How does the timing of UMAD1/TSG101 compare with CEP55?
To the best of our knowledge, our antibody against UMAD1 is the only one available, and this antibody does not work for immunofluorescence. It is therefore not feasible to show the localisation of the endogenous UMAD1. However, it is worth highlighting that our YFP-UMAD1 construct is functional, as shown in Figure 3C, thus supporting the relevance of the midbody localisation of this construct.
Our data shows that UMAD1 and TSG101 are recruited together to the midbody. Elia et al (PMID: 21383202) have shown that CEP55 is recruited earlier than TSG101 to the midbody. We therefore infer that CEP55 is recruited earlier than the TSG101/UMAD1 complex to the midbody.

Figure 5. The description of Figure 5A in the text is somewhat confusing. “Demonstrating that UMAD1 acts downstream (of?) CEP55 and ESCRT”? If UMAD1 is an ESCRT1 component then UMAD1 acts alongside rather than downstream of TSG101. This section could be rephrased. The TSG101 blot is poor quality and very tightly cropped. The panel labelling in the figures (Merge and YFP only) is a little confusing and it would be helpful to reference tubulin in the label. The merges are inconsistent (R-G; M-G) and magenta-green is preferable.

© 2023. Published by The Company of Biologists under the terms of the Creative Commons Attribution License (https://creativecommons.org/licenses/by/4.0/).

This sentence is now changed to "…demonstrating that UMAD1 acts downstream of CEP55 and its recruitment to the midbody requires other ESCRT-I subunits".
A new set of blots is now included in this figure, including an improved TSG101 blot.
The labelling of the microscopy figure has been modified as requested. We favour keeping the current pseudo colouring scheme for the microscopy data as it differentiates between mCherry-tubulin (Red) and SiR-tubulin (Magenta), which is consistent throughout the manuscript.

Figure 6A. It would be helpful to see a blot of the CHMP4 in these experiments, to show whether CHMP4B levels are affected by the conditions but also to confirm that CHMP4B-GFP levels are close to endogenous CHMP4B levels. The IF panels need to be labelled, and green-magenta is preferable.
Blots for CHMP4B, ALIX and Hsp90 are now included in this figure. A new related supplementary figure (S4) includes western blots showing that CHMP4B-L-GFP is expressed at lower level than the endogenous CHMP4B. This is indicated by the lack of detection of CHMP4B-L-GFP with the anti-CHMP4B antibody, whilst this fusion is clearly detected by the anti-GFP antibody in the same cell lysates.
The immunofluorescence panels are labelled as requested. As explained above, we have kept the colour scheme in this panel for consistency across the manuscript.

Figure 6B. Can statistical analysis be performed please?
The statistical analysis of this figure is now provided in the figure legend.

Reviewer 2 Advance Summary and Potential Significance to Field:
Review UMAD1, Glover, JCS
In the submitted manuscript the authors identify UMAD1 as a a new ESCRT-I component and characterize its role in cytokinetic abscission. Moreover, they provide evidence to suggest that UMAD1 is an abscission specific ESCRT-I component. While ESCRTs are known to drive numerous processes in cells, the basis for their specific recruitment to the site of action is still largely unknown. Additionally, while the ESCRT-III and VPS4 ESCRT subcomplexes have been extensively studied in cells our knowledge on the function and regulation of the early ESCRT subcomplexes (ESCRT-I and II) inside cells is limited. Therefore, the findings presented in the manuscript advance our understanding of ESCRTs, in general, and of ESCRT-mediated cytokinetic abscission, in particular. The manuscript is concise, well written and most conclusions are fully supported by the experimental results. I do have several comments which should be addressed prior to publication.
The thank the reviewer for these enthusiastic comments. We are confident that the constructive points raised by this reviewer have been addressed in the revised manuscript.

Reviewer 2 Comments for the Author:
1.My main concern in the manuscript is the FRAP experiments and their interpretation. First, on the technical side, it is not clear to me why the CHMP4B recovery is less than 0.5 (in the Mierzwa 2017 publication the reported recovery is close to 1). This difference should be explained. Second, the results obtained in the delta UMAD1 compared to siRNA Alix, suggests that most of the phenotype is contributed by depleting Alix rather than by depleting UMAD. I understand that depleting both proteins is essential for getting an abscission phenotype due to compensation between the two pathways, but I feel that concluding from these data a connection between UMAD and protein exchange in the ESCRT-III filament is a stretch. I recommend toning down the title and main text in relevant places, modifying the model to avoid inferring a direct effect and explicitly refer to this issue in the discussion. Alternatively, more experiments should be performed to substantiate the connection between UMAD and CHMP4B exchange.
We agree with the reviewer that there are some minor differences in the FRAP data compared to Mierzwa et al, but these can be easily rationalised as the experimental conditions are not fully comparable. The main difference with our FRAP experiments is the level of expression of the tagged CHMP4B construct. As we now show in the revised manuscript (Figure S4), our CHMP4B-L-GFP construct is undetectable using anti-CHMP4B antibodies, thus implying that the vast majority of the "CHMP4B" available in these cells is the endogenous, untagged, form of the protein. Mierzwa et al perform similar expression analysis, but their cells express much higher levels of the mmCHMP4B-LAP construct than the endogenous CHMP4B. In other words, the proportion of tagged CHMP4B vs endogenous CHMP4B in our cells is dramatically lower. In this context, the dynamics of the fluorescent signal recovery is expected to be different in our cells, as CHMP4B-L-GFP must compete with an excess of untagged protein for incorporation into the ESCRT-III polymers. It is

© 2023. Published by The Company of Biologists under the terms of the Creative Commons Attribution License (https://creativecommons.org/licenses/by/4.0/).

therefore inappropriate to directly compare the FRAP dynamics from cell lines that express different levels of tagged CHMP4B, and the only relevant comparison is between the different conditions within the same cell lines.

We share the reviewer's view on the contribution of UMAD1 to the dynamic turnover of ESCRT-III, as shown by our assessment of this phenotype in the results section: "the turnover of CHMP4B-L-GFP in control-treated cells was comparable between $HeLa^{WT}$ and $HeLa^{\Delta UMAD1}$ cells". Nonetheless, we take this comment on board and we have modified the manuscript title to "UMAD1 contributes to ESCRT-III dynamic subunit turnover during cytokinetic abscission". We have also modified the model in figure 6D to highlight that ESCRT-III dynamic exchange is inhibited when the expression of both ALIX and UMAD1 are reduced. Lastly, we have modified the last sentence in the discussion to highlight that the effects of UMAD1 and ALIX on ESCRT-III dynamic exchange may be indirect.

2.The authors nicely mapped the interactions with proteins within the ESCRT-I subcomplex and with CEP55, which is an upstream component that recruits ESCRT-I. What about interactions with proteins of the downstream ESCRT-II subcomplex? It will be good to provide experimental data on that or at least to discuss ESCRT-II interactions somewhere in the text.
Our proteomics experiments did not show any hint that UMAD1 may interact with ESCRT-II. Therefore, we have not pursued this avenue. A sentence in the results section has been added to mention the lack of evidence supporting the association between UMAD1 and ESCRT-II.

Minor comments:
1.Fig. 2 – It will be nice to add a scheme summarizing the interactions observed for UMAD1. It is a bit hard to follow.
We believe the suggested diagram would be redundant as the summary of these interactions is included in the model shown in Figure 6D.

2.Fig. 2D – grey levels in anti-GFP and anti-VPS37C gels appear too narrow. Bands are missing the typical haze surrounding the bands as seen in the other panels and throughout the figure. Please replace with less filtered versions.
As explained above, the intense signal in these blots gives the appearance of highly contrasted bands. The brightness of these blots is now adjusted to minimise this effect.

3.Fig. 3 A-B – Protein loading controls point to a difference in total protein levels. It should not affect the conclusions, but it is important to indicate whether this was taken into account when calculating the percentage of expressed proteins in the different conditions and it should be noted in figure legends. Alternatively, the gels can be replaced with gels in which the total protein levels are similar to avoid confusion.
As suggested by the reviewer, new and improved blots are now included in this figure.

4.Fig. 3 C – Image quality is poor. Please improve to make sure the intercellular bridge can be clearly seen. Also, what does the yellow arrow indicate? Arrows are not specified in Fig. legend.
We realise these images were too small. We have now enlarged them to improve the clarity. The midbody is shown with white arrowheads whilst the yellow arrowhead denotes abscission failure. This information is now included in the figure legend.

5.Fig 3 D – It should be clearly indicated in the cumulative plot that only cells that successfully completed abscission were quantified. It is mentioned in the Fig. legend but given the multinucleated cells phenotype I think it is important to specifically indicate that on the Y axis of the plot.
The label of the Y axis in figure 3D is now modified to indicate it represents the "% of successful cell divisions".

6.I think it is better to show STDEV rather than SEM.
We favour the use of Standard Error of Mean (SEM) because it quantifies how precisely you know the true mean of the population and it is closely related to the confidence interval and P value, considering both the value of the SD and the sample size.

© 2023. Published by The Company of Biologists under the terms of the Creative Commons Attribution License (https://creativecommons.org/licenses/by/4.0/).

<u>Second decision letter</u>

MS ID#: JOCES/2023/261097

MS TITLE: UMAD1 contributes to ESCRT-III dynamic subunit turnover during cytokinetic abscission

AUTHORS: James Glover, Edward J Scourfield, Leandro N Ventimiglia, Xiaoping Yang, Steven Lynham, Monica Agromayor, and Juan Martin-Serrano
ARTICLE TYPE: Research Article

I am happy to tell you that your manuscript has been accepted for publication in Journal of Cell Science, pending standard ethics checks.

<u>Reviewer 1</u>

Advance summary and potential significance to field

The authors have addressed all of my comments satisfactorily. This is a great piece of work that has been tidied up sufficiently for publication in JCS.

Comments for the author

NA

<u>Reviewer 2</u>

Advance summary and potential significance to field

The authors have fully addressed my comments.
I recommend publication.

Comments for the author

The authors have fully addressed my comments.
I recommend publication.

© 2023. Published by The Company of Biologists under the terms of the Creative Commons Attribution License (https://creativecommons.org/licenses/by/4.0/).
